# Word-color association from large-scale online data: Methodology and applications

**DOI:** 10.1371/journal.pone.0360407

**Published:** 2026-10-16

**Authors:** Sungpil Wang, Juyong M. Park

**Affiliations:** 1 Graduate School of Culture Technology, Korea Advanced Institute of Science and Technology, Daejeon, Republic of Korea; 2 School of Computational Sciences, Korea Institute for Advanced Study, Seoul, Republic of Korea; Southwest University, CHINA

## Abstract

Words and colors are intimately associated in humans, a sign of the close relationship between verbal, linguistic expression and cognition. Word-color association therefore plays an important role in areas of communication such as marketing and literature. Traditional efforts to build a general, scalable, and quantitative mapping of word-color association have often relied on surveys, experiencing difficulties in curating large-scale data sets, enrolling sufficient participants, and excising potential inherent researcher biases in the choice of the words and the colors to be mapped. Even the most recent computational efforts based on Large Language Models (LLMs) do not overcome these problems due to their near-complete lack of transparency in data and methodology that results in low confidence, consistency, and reliability. Here we propose a framework that overcomes these limitations via the use of freely accessible large-scale web image data, and fully transparent and reproducible methods that allow one to build color associations for almost any given word. We also showcase the methodology in action through analyses of color usage in corporate branding and 20th-century Anglo-American poetry. These show that our framework can help form new answers to and perspectives on long-standing questions in communication. Additionally, our framework by design can be readily adopted for use in any language supported by a web image search engine, and its code is freely available in open source; we therefore expect it to contribute towards achieving a universal, multilingual understanding of word-color association.

## Introduction

Word-color association represents a powerful and useful connection between language and human perception. It is not merely random or completely personal, but well known to exhibit robust and consistent patterns shaped by culture and social contexts [1,2]. The most familiar and strongly universal examples include ‘money’ associated with green, and ‘love’ associated with red and pink. Such universality allows word-color associations to serve a practical function in communication of enhancing the clarity and efficiency of a message, thereby reducing cognitive load and facilitating the arousal of its intended emotional responses [3,4]. This has led to interesting uses in marketing where corporations employ characteristic, immediately recognizable colors to establish their identities and form bonds with consumers [5,6], a well-known example being the global freight giant FedEx now firmly characterized and recognized by the combination of orange and purple [7,8]. Another area where word-color association plays an important role is literature where ‘color symbolism’–-employing colors to reference or signify often deep cultural, historical, and social attitudes, values, and meanings–-plays an essential role [9–11].

The importance and ubiquity of word-color association have motivated multiple attempts to tabulate a robust mapping between words and colors for research and other purposes. In one line of approach, human participants are asked to link a group of presented words with one of the predefined set of color options [12–15]; In another, the problem is tackled lexically–-either by querying linguistic resources such as WordNet for color terms associated with a word [16], or by computing co-occurrence statistics with color words via measures such as Pointwise Mutual Information (PMI) or Latent Semantic Analysis (LSA) [17]; In a third, numerical color values are extracted directly from collections of web images from Flickr or Google image search, the closest of the basic colors being assigned to the given query word [1,18,19]. And most recently, Large Language Models (LLMs) have been used to try to directly identify the associated colors from prompt queries [20].

These approaches share common shortcomings that limit their capability to capture the complexity of word-color association and instill confidence in the users: Survey-based approaches feature small participant pools and present only finite, arbitrarily curated word sets [12–15]; Lexical and linguistic-similarity approaches depend on explicit color terms appearing in dictionary entries or in word co-occurrence tables, leaving out those words that lack such linguistic links which then can be overcome only by indirect inference [16,17]; And web image-based approaches to date have often extracted a single dominant color from a returned image, discarding the complete, rich color information that encodes the full complexity of human visual perception, or have operated in the simplistic RGB color space known for not aligning optimally with true human perception that can introduce researcher bias when assigning semantic labels to numeric color values [1,18,21,22]. Even in some cases where those limitations have been addressed, typical outputs are given in the form of regions of a perceptual color space that is not as easy to grasp as a user-friendly weighted combination of named colors such as ‘red’ or ‘blue’ familiar to humans could be [19,23]. Finally, LLMs are essentially ‘black boxes’ with a total lack of transparency regarding data and method, rendering the so-called ‘answers’ provided impossible to validate with a sufficient level of confidence [20] and thus unfit for use in a rigorous investigation.

In this work we present a framework for establishing word-color association that aims to remedy those problems stemming from insufficient data, human researcher bias, unnecessary loss of color information, and lack of methodological transparency. Specifically, our method overcomes these issues by utilizing a publicly accessible large-scale web image dataset retrieved for an input word as the search term, and representing it as a 10-dimensional vector of base color coordinates that can simply be added (superposed) to find the associated color vector of a group of words (e.g., a document). Additionally, the color coordinates are in the CIELch color space consistent with human perception unlike the RGB, minimizing *ad hoc* biases that allows us to establish a more general word-color association that better captures its complexity. Our methodology is also fully transparent unlike LLM-based methods, and its workflow is made available as a free, open-source module (See S1 File) for the public to use.

We demonstrate our method by applying it to the two aforementioned areas of communication where color plays an important role, corporate branding and literature. In corporate branding, color has always been a core element in logo and product design as well as advertisements, and is now becoming even more consequential in the digital domain where visuals are central [24]. Furthermore, it has been reported that color-use strategies differ from industry sector to industry sector [25] depending on the image that each industry wishes to project onto the clients, implying that we need to go beyond simply cataloguing the ‘official’ colors of the brands to understand it properly; this would involve extracting sets of colors–-not simply the most dominant color–-from a wide range of images associated with a brand, which our method is well suited for. As for literature, the use of colors as symbols that convey meanings shaped by cultural traditions, social contexts, and collective experiences is called ‘color symbolism’, also known to vary widely across different cultures [26,27]. As such, color symbolism in a piece of writing can take the form of a direct and explicit use of a color term, or an indirect and implicit use of a word referencing culturally significant objects or images associated with the color that the writer has intended [28]. In either case, traditional studies of color symbolism in literature have relied on the so-called ‘close reading’ technique where researchers must manually identify explicit references to colors word by word, having to personally judge the symbolic meaning of implicit references in a given corpus (likely small, as it has to be read by humans in a given amount of time) [29,30]. This also suffers from a lack of reproducibility due to a total reliance on the subjectivity of the ‘close reader’ and the limited scale of data that can be processed. This problem has led to a heightened interest in a contrasting method called ‘distant reading’ that uses quantitative, computational methods on large-scale corpora that have become increasingly accessible due to advances in digital humanities [31–33]. Applying our method to study color symbolism in literary texts can also be considered an instance of distant reading, allowing us to overcome the limited scale of human-chosen color terms and the subjective judgments of the reader [34]. In this paper, we analyze the *complete* corpora of two prominent 20th-century Anglo-American poets renowned for their use of color in their works but widely understood to have taken contrasting approaches to it, Amy Lowell (1874–1925) and Georgia Douglas Johnson (1880–1966).

## Materials and methods

Our framework is a multi-step process consisting of (1) Web image retrieval and preprocessing, (2) Conversion of pixels to the CIELch space, and (3) Mapping of the color coordinate distributions into chromatic and achromatic base colors to establish the desired word-color association.

### Web image retrieval, preprocessing, and color conversion to the CIELch space

We start with the images retrieved from a Google search for the word of interest. This is based on prior research showing that the images indexed by major search engines effectively capture the visual semantics of human language [35,36]. Those images are uploaded, tagged, and ranked by a global user base, serving as an implicit yet robust aggregation of collective visual knowledge [37–39]. Modern web-search rankings are designed to aggregate various correlational factors such as link structure [40] and large-scale user behavior [41,42]. This principle has been extended to image search via click-based [43] and visual-similarity [44] reranking; Google’s own image-search guidance documents various factors such as image quality and contextual relevance (e.g., alternative texts and surrounding page content) as factors affecting ranking [45]. The order of the images returned from a search query is therefore not random, but reflective of the rankings.

To mitigate the potential user biases stemming from individual search histories or browser cookies, all queries are conducted in the *incognito* mode with these deleted and disabled. To further ensure a consistent and reproducible search setting, we explicitly standardize the regional (i.e., *gl = us*) and the language (i.e., *hl = en*) parameters to set up a depersonalized baseline that would return the broadest aggregated search outputs [46].

The top viewport (the first page of returned images) of the Google image search results would then capture the most highly ranked subset of the visual signals associated with the query–-what the users typically encounter and interact with. Still, we find that even immediately lower viewports (close but later pages) are largely consistent with the highest ranked ones: across the pages 1–10 of the search results, the pairwise cosine between any two viewports averages to ≥0.94 while its mean decays slowly with page distance (Pearson r=−0.98). The color vector of the top viewport also agrees with the aggregates of *K* randomly sampled deeper viewports with median cosine ≥0.97 for every K∈{2,...,9} (S1 Fig), indicating an abundance of relevant images.

In cases where a word carries multiple senses (polysemy), a user would need to be more specific to reduce ambiguity. To see the effects of polysemy, we tested 30 common English nouns by running a simple undisambiguated (sense-aggregated) query and a sense-disambiguated one using the WordNet noun synset (the sense-adding word is chosen via a fixed rule; see S2 Fig caption) through the standard pipeline, and compared the resulting 10-dimensional color vectors. The spread among a word’s sense vectors indeed grows with the number of its senses (Spearman ρ=0.38, *p* = 0.038, *N* = 30; S2 Fig), suggesting that the method is indeed able to return distinct results for the different senses specified by the user.

There are two scenarios in which the method may not work properly or be sufficiently meaningful, i.e., when the words are not adequately represented (i.e., too few returned images), and when the images are predominantly meaningless ‘text images’–the search term itself in a generated image format. For the first scenario, we used the Bing Image Downloader module [47] to filter out those with fewer than 30 images. For the second scenario, the Pytesseract Python package [48] was applied to the ten leading returned images to detect such text images; when more than three images were deemed to be texts, the returned images underwent manual inspection. Fig 1 shows the image retrieval and color conversion process for an example search term ‘moss’.

**Fig 1:**
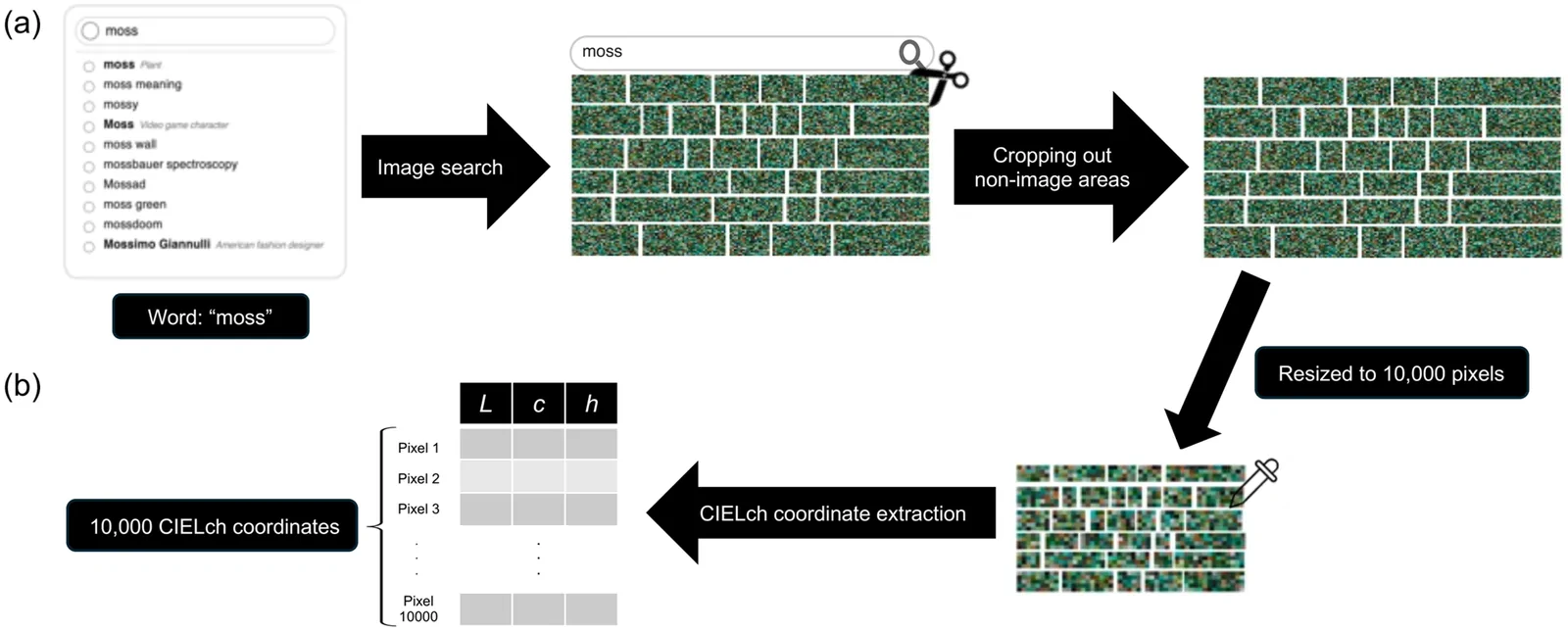
Word Search, Image Retrieval, and Color Extraction. A query term (“moss” in this example) is submitted to Google image search. The top-ranked results are returned as a screenshot of the results page, a grid of individual images separated by white gutters. The screenshot is then resized to 10 000 pixels, and each pixel is converted to a CIELch coordinate for decomposition to base colors. (a) Screenshots of retrieved images are preprocessed and resized to 10 000 pixels. (b) Each pixel of the screenshots is stored as a CIELch coordinate (*L*,*c*,*h*).

To minimize computational load and to avoid problems such as extreme variations in resolution and dynamically-generated contents, the returned images are not individually stored, but collectively as screenshots of the search results page [49–51] automated via the Python Selenium package [52]. Each screenshot is then resized to 10 000 pixels using the Python Pillow library, maintaining the original aspect ratios and the colors as close to the original as possible using the Lanczos and antialiasing filters [53,54]. The unwanted pixels such as website logos and the white gutters (divider spaces) between the images (see S3 Fig) are then cropped and trimmed out.

Finally, each pixel in the preprocessed screenshots is stored as an (*L*,*c*,*h*) coordinate in the CIELch color space, converted from the original RGB space using the scikit-image package. CIELch, the polar representation of the CIELAB space of CIE (International Commission on Illumination), is the preferred choice for accurate color comparison studies (including our work) due to its capacity to encode the full range of human daylight vision and color perception [55,56]. Each component of (*L*,*c*,*h*) means the following:

- **Lightness** L∈[0,100] represents the brightness of a pixel.
- **Chroma** c∈[0,133.80] represents the color saturation or intensity within the sRGB gamut [57].
- **Hue** $h\in [0^{\circ },360^{\circ })$ represents the perceived color class, i.e., what humans perceive as red, blue, *etc.*, from the color wheel (hence the degree °).

### Mapping color coordinates to chromatic and achromatic base colors

While the (*L*,*c*,*h*) coordinate distribution thus obtained contains the complete original information on the associated colors of the input word, it is difficult for humans to visualize or intuitively understand the numerical values. It would, therefore, be intuitive and pragmatic to express the (*L*,*c*,*h*) distribution using common, familiar color names, e.g., how much ‘blue’ it contains, how much ‘red’ it contains, *etc.* In this manner, the stated goal of a multidimensional word-color association is maintained while achieving an intuitive interpretability of the colors.

As the ‘base colors’, we use ten of the eleven fundamental colors found to be universal across many cultures and civilizations [58]–seven chromatic and three achromatic (grayscale):

- Chromatic bases: Red, Orange, Yellow, Green, Blue, Purple, and Pink.
- Achromatic bases: Black, White, and Gray.

Brown from Ref [58] was excluded in this work to clearly distinguish between the two classes, as brown spans both categories [59,60].

The fundamental idea is to first establish the color coordinates of these color names by using them as the search terms in Fig 1, then compute the overlap between these (specifically, the hue *h* for the chromatic bases and the lightness *L* for the achromatic bases) and the coordinates of the word of interest. To maximize the coverage in determining the color coordinates of the base colors, we include the synonyms of the colors as they appear in the Oxford American Writer’s Thesaurus [61,62] (four to five for each color, chosen randomly if more than five are listed in the thesaurus included in the Apple OSX operating system). For instance, the color distribution of the base color Red is the union of those of ‘red’, ‘scarlet’, ‘vermilion’, ‘ruby’, and ‘carmine’ (See S4 Fig). Table 1 shows the synonyms of the base colors. Note that the base color names are capitalized as a convention in this work to distinguish them from the individual search terms (e.g., ‘Red’ as the union of all synonyms versus the literal ‘red’).

**Table 1:** Base colors and their synonyms. Synonyms were selected from the Oxford American Writer’s Thesaurus [61,62]. Base color names are capitalized to distinguish them from the literal search terms.

Base color	Synonyms
Red	*red, carmine, ruby, scarlet, vermilion*
Orange	*orange, apricot, marmalade, orangish, tangerine*
Yellow	*yellow, gold, golden, yellowish, yellowy*
Green	*green, greenery, greenish, leafy, verdant*
Blue	*blue, azure, cerulean, cobalt, ultramarine*
Purple	*purple, amethyst, purplish, purply, violet*
Pink	*pink, blushing, rose, rosy, shell pink*
Black	*black, blackish, jet black, pitch black, pitch dark*
Gray	*gray, slate gray, silver, silvery, smoke gray*
White	*white, chalk white, milk white, milky white, snowy white*

The distributions of *h* (for chromatic) and *L* (for achromatic) of the base colors are shown in Fig 2(a) (the curves were smoothed using Ref [63]; See S5 Fig for the detailed frequency distributions of individual color synonyms). We see that each base color dominates one uninterrupted range: for instance, the base color Red is the dominant one in $h\in [19.33^{\circ },45.68^{\circ })$, and Gray in L∈[41.65,82.90), *etc.* The detailed ranges of the base colors are also given in Table 2. Using the table we can now conveniently represent a word’s color association as a set of ten values that are the fractions of its *h* and *L* components that fall into each interval. As an example, in Fig 2(b) we show the color association of the word ‘lilac’, which has the largest fraction (0.4306) of its *h* in Purple, followed by 0.2458 in Green, agreeing well with our intuition that the color of the flower’s petals (Purple) and leaves (Green) would be the most strongly associated, but now with a clearly defined quantitative association value; The largest achromatic component, on the other hand, is Gray with 0.6151.

**Table 2:** The base color intervals (hue *h* for the chromatic base colors, and lightness *L* for the achromatic base colors).

Base color	Range (*h* for chromatic, and *L* for achromatic colors)
Red	$h\in [19.33^{\circ },\;45.68^{\circ })$
Orange	$h\in [45.68^{\circ },\;80.91^{\circ })$
Yellow	$h\in [80.91^{\circ },\;120.18^{\circ })$
Green	$h\in [120.18^{\circ },\;203.28^{\circ })$
Blue	$h\in [203.28^{\circ },\;293.87^{\circ })$
Purple	$h\in [293.87^{\circ },\;331.25^{\circ })$
Pink	$h\in [331.25^{\circ },\;19.33^{\circ })$
Black	L∈[0,41.65)
Gray	L∈[41.65,82.90)
White	L∈[82.90,100]

**Fig 2:**
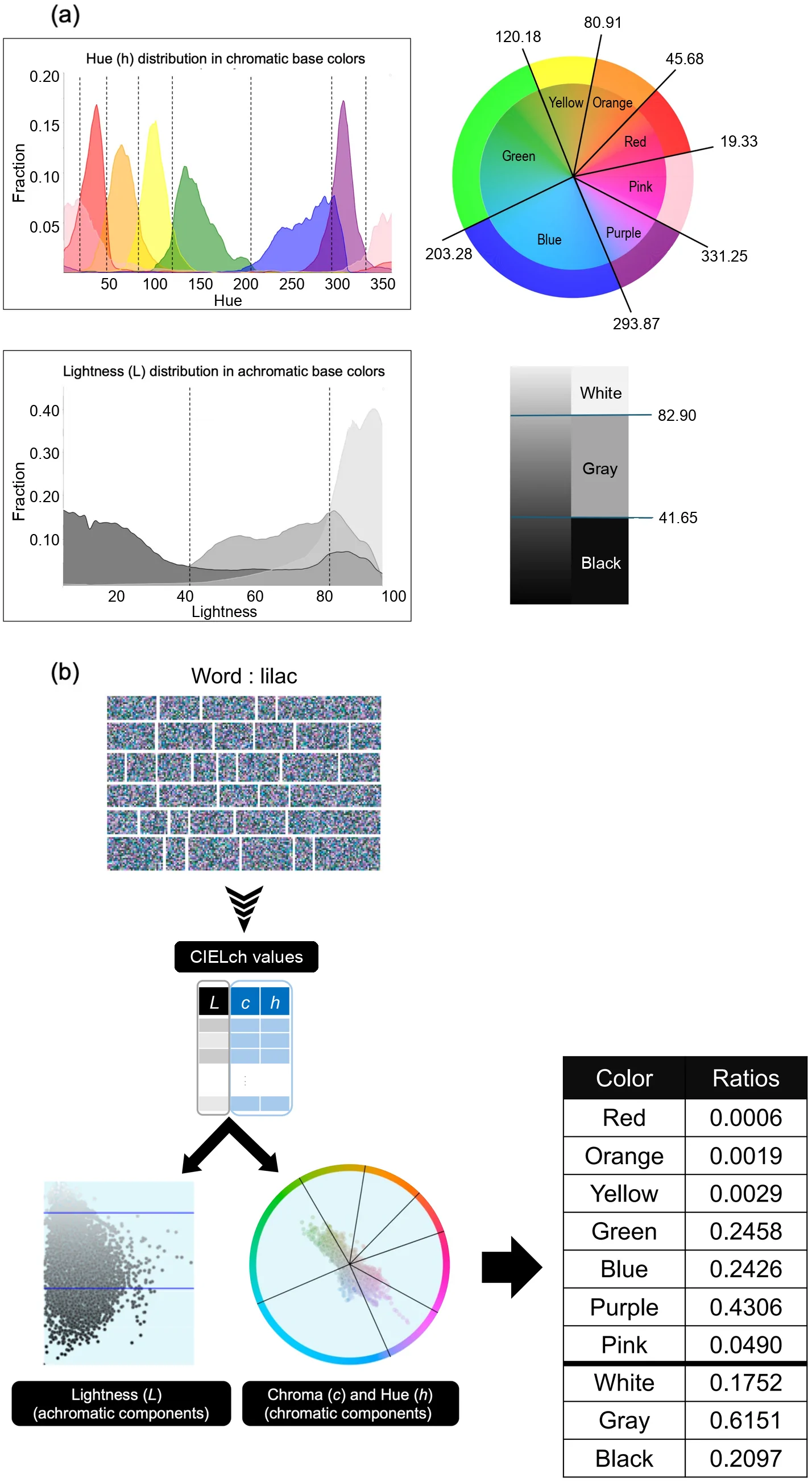
Chromatic and Achromatic Base Colors and Multidimensional Word-Color Association. (a) Hue *h* (for chromatic base colors) and lightness *L* (for achromatic base colors) distributions. Each base color shows a single, uninterrupted range where it dominates; We then divide the full range of each into the seven *h* and the three *L* intervals representing the corresponding base colors. (b) The final multidimensional word-color association of a search term is the set of ten values equal to the fraction of its pixels that belong in each interval.

In the example of ‘lilac’ (with pixels extracted from the screenshot of the search results), the most dominant chromatic colors are Purple and Green, obviously representing the flower’s petals and leaves.

Finally, we note that while *c* itself is not used in Fig 2(b) *per se*, it is involved in the process of filtering out certain chromatic colors: Due to the uncertainty in the hue *h* of a pixel with too low a chroma *c* [64–66], pixels with *c* below a threshold of *c*_0_ = 15.93 were not considered for hue; only their lightness *L* was used. The threshold *c*_0_ was determined from an analysis of the chroma distributions of the fifteen synonyms of the achromatic base terms Black, Gray, and White given in Table 1 (also shown in S6 Fig). In each case we measured the *c* values below which 95% of the returned pixels are included, then took the highest value among them, *c*_0_ = 15.93, as the threshold. *c*_0_ can thus be considered the minimum chroma separating the chromatic and the achromatic colors.

For validation purposes, our framework was compared with four prior approaches as benchmarks that span the range of methodological approaches outlined in the Introduction and that provide public datasets, codes, or per-word vectors: the NRC Word-Colour Association Lexicon [12] (survey-based forced-choice categorical lexicon), Rathore et al. [23] (survey-based graded slider dataset with image-statistics integration), a Setlur & Stone-style pipeline [16] (lexical), and comp-syn [19,67] (image-based color embedding with publicly released per-word vectors). Other approaches cited in the Introduction either belong to a methodological family with one of these and would be largely redundant (e.g., additional survey-based lexicons [13–15]) or do not release per-word color data in a form that allows direct comparison (e.g., Lindner et al. [18] depends on Flickr’s closed API for image acquisition; LLM-based methods [20] do not at all support reproducibility for methodological comparison). Per-benchmark methodology, comparison metric, results, and the limitations of the various methods are summarized in S1 Table; see S7–S10 Figs for the individual comparisons. Also note that, because no ground-truth word-color mapping can exist, these comparisons are primarily a measure of consistency across distinct methodologies rather than accuracy. Our method shows moderate levels of agreement with each benchmark, but can produce a 1:*N* additive distribution over base colors that single-color baselines cannot (e.g., *snowflake* is distributed across blue, white, and gray rather than collapsed to ‘white’ alone).

### Corporate logos and color symbolism in literature

For corporate branding and logo analysis, we measure the correlation between the official logos of corporations and the brand-associated web images, and examine whether the correlations are industry sector-dependent. For corporate branding analysis we study the world’s top 100 publicly traded companies (as of February 2025) whose names and industry sector classifications were retrieved from TradingView (https://www.tradingview.com/), whereas their official logos were obtained from Wikipedia (https://www.wikipedia.org/). Each company’s full registered name as listed on TradingView is used as the query to Google Image Search so that the resulting color profile reflects the brand-associated visual content publicly indexed under the corporate name–-e.g., logos, products, packaging, advertisements, editorial usage, photos of executives, and company events.

For color symbolism analysis in literature, we adopt a common modus operandi in digital humanities of applying the contemporary computational perspective to historical literary material: Each word’s associated colors reflect the visual content *currently* indexed for that word across large-scale web imagery. This approach serves two purposes: First, it characterizes how today’s visual semantics engage the language of these texts–-a present-day reception of direct interest to today’s readers, teachers, and scholars who read these early-twentieth-century poets [68,69]; Second, it establishes a reproducible baseline for future period-specific analyses [70–72]. Because both poets are read against the same present-day data, the differences between them reflect their different vocabulary choices, rather than period-specific visual cultures.

Specifically, we measure the overall color associations in the complete works of Amy Lowell and Georgia Douglas Johnson, freely available from Project Gutenberg and elsewhere in PDF format (S2 Table). The texts are converted to a computer-readable form via the Tesseract Optical Character Recognition (OCR) package [48,73], after which Python’s pandas and NLTK libraries [74] are used for stopwords and special character removal, uppercase-to-lowercase conversion, retention of words potentially relevant to imagery (nouns, verbs, adjectives, and adverbs), and lemmatization into base forms (elimination of morphological variations) [75]. Each poet’s corpus is treated as a bag-of-words (BoW) so that its associated color is obtained by superposing (summing up) its constituent words’ color vectors and then normalizing it. Also, we note that each word’s color vector–-whether the word is an explicit chromatic term (Table 1) or not–-contributes equally to its poet’s bag-of-words sum. While this universality is a core feature of our method, it would be instructive to further assess the impact of this choice, so we gave varying weights α∈[0.1,1.0] to general (non-chromatic) words and traced whether the sign and the ordering of each color’s $\Delta =f^{L}-f^{J}$ were preserved throughout (S11 Fig). To test whether each color’s $\Delta_{c}$ reflects a statistically significant contrast between the two corpora, we used a permutation null test [76,77]: The pooled Lowell–Johnson content-word lexicon was randomly partitioned into two size-matched corpora (*B* = 10 000 partitions), and the observed $\Delta_{c}$ was compared to the resulting null distribution to obtain a two-sided *p*-value, defined as the fraction of null draws with $\left|\Delta_{c}^{null}|\geq |\Delta_{c}^{obs}\right|$ (S12 Fig).

## Results and discussion

### Corporate branding and industry sector

We first calculate how strongly aligned the colors of the official logos and the associated images on the web of the top 100 global companies are using cosine similarity, as shown in Fig 3 (the complete, raw cosine similarity values for all 100 companies are provided in S4 Table). The mean cosine similarity of each industry sector is visualized in Fig 4 where we see that the Communications, Energy & Minerals, Retail, Technology Services, and Producer Manufacturing sectors show a strong alignment, whereas the so-called fast-moving consumer goods (FMCG) and customer-facing services of non-durables and consumer services exhibit a weak alignment. This trend is consistent with the aforementioned prior findings that consumer-adjacent industries tend to employ more diverse visual strategies that can distinguish between the sub-brands than durable and consumer-distant industries that tend to maintain more unified visual identities [78].

**Fig 3:**
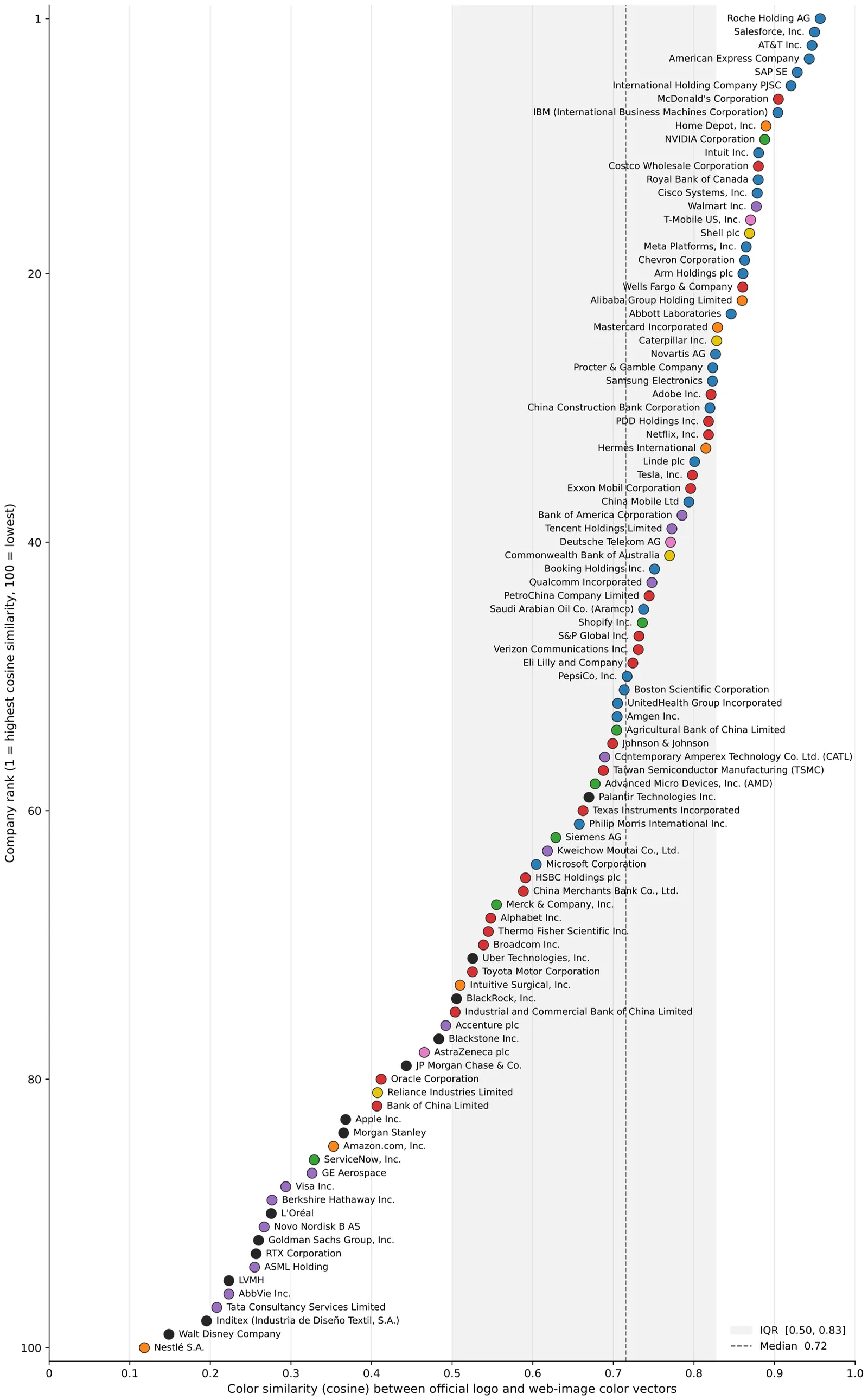
Color alignment between corporate logos and web images. The top 100 companies arranged by cosine similarity between their official logos and the screenshot of the web search results returned on their full registered names. Each company is shown as a single point on the cosine-similarity axis at its corresponding rank (1 = the highest similarity, 100 = the lowest), with the dot’s color set to the extracted dominant base color of the official logo. The vertical shaded band marks the interquartile range while the dashed line marks the median across the 100 companies.

**Fig 4:**
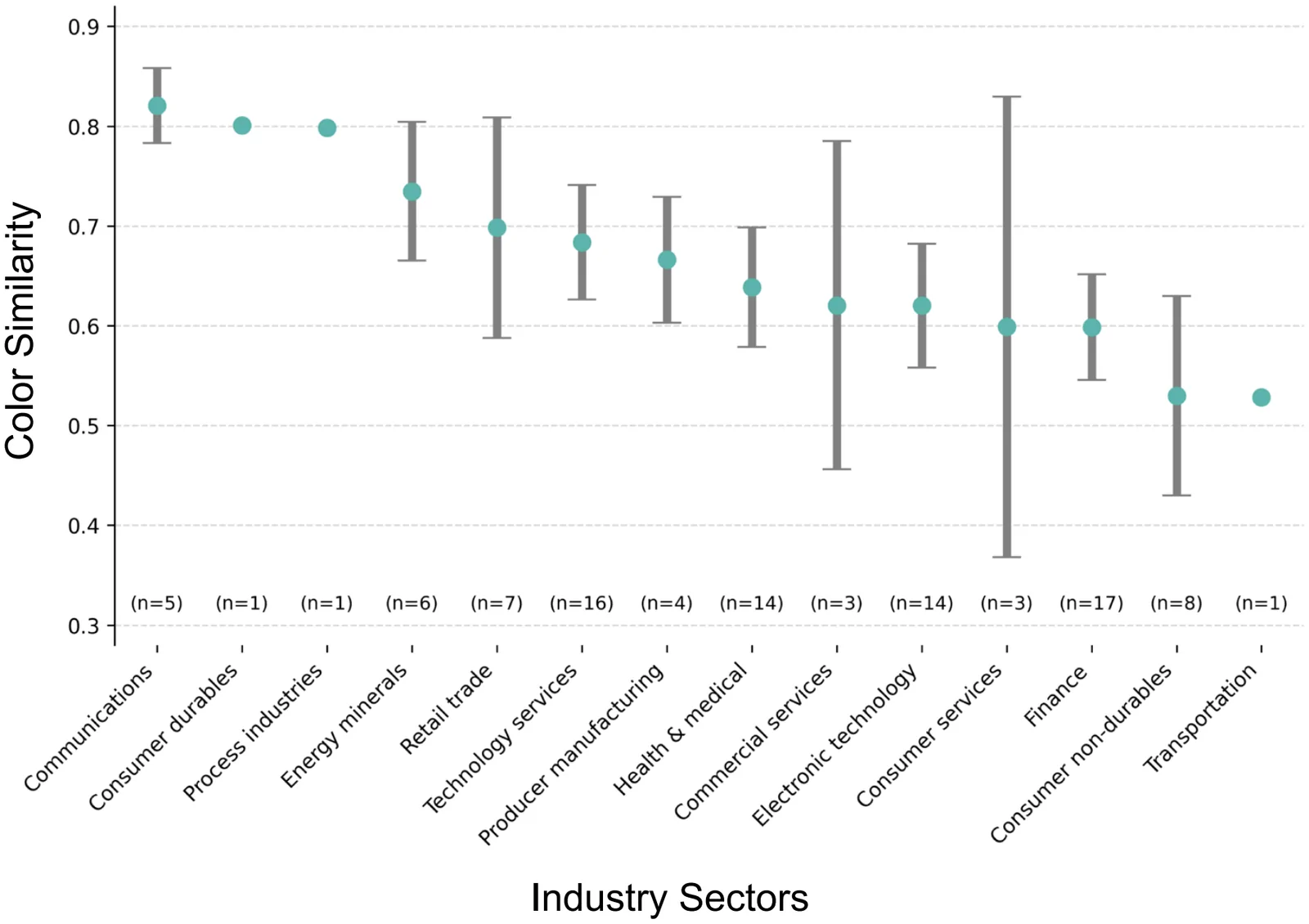
Differences in color alignment depending on the industry sector. The mean cosine similarity and standard errors are shown, arranged by the mean. The number of corporations (*n*) in each sector is indicated. The sector definitions are listed in S3 Table.

For illustrative purposes, the associated colors of two of the most strongly aligned brands and industries (AT&T from communications and Caterpillar from producer manufacturing) and two of the most weakly aligned (Walt Disney from consumer services, and Nestlé, the weakest among the companies and a well-known maker of diverse food and packaging) are detailed in Fig 5. There we see that the logos of AT&T and Caterpillar form a strong identity through uniformity, but Walt Disney and Nestlé are quite the opposite, with many colors used in their diverse consumer-adjacent products. From these results, we can see that our method allows us to go beyond many previous studies that were limited to individual product categories or specific industry settings [6,25,79,80] through a systematic, large-scale analysis of accessible data on the color associations of a wide range of brands.

**Fig 5:**
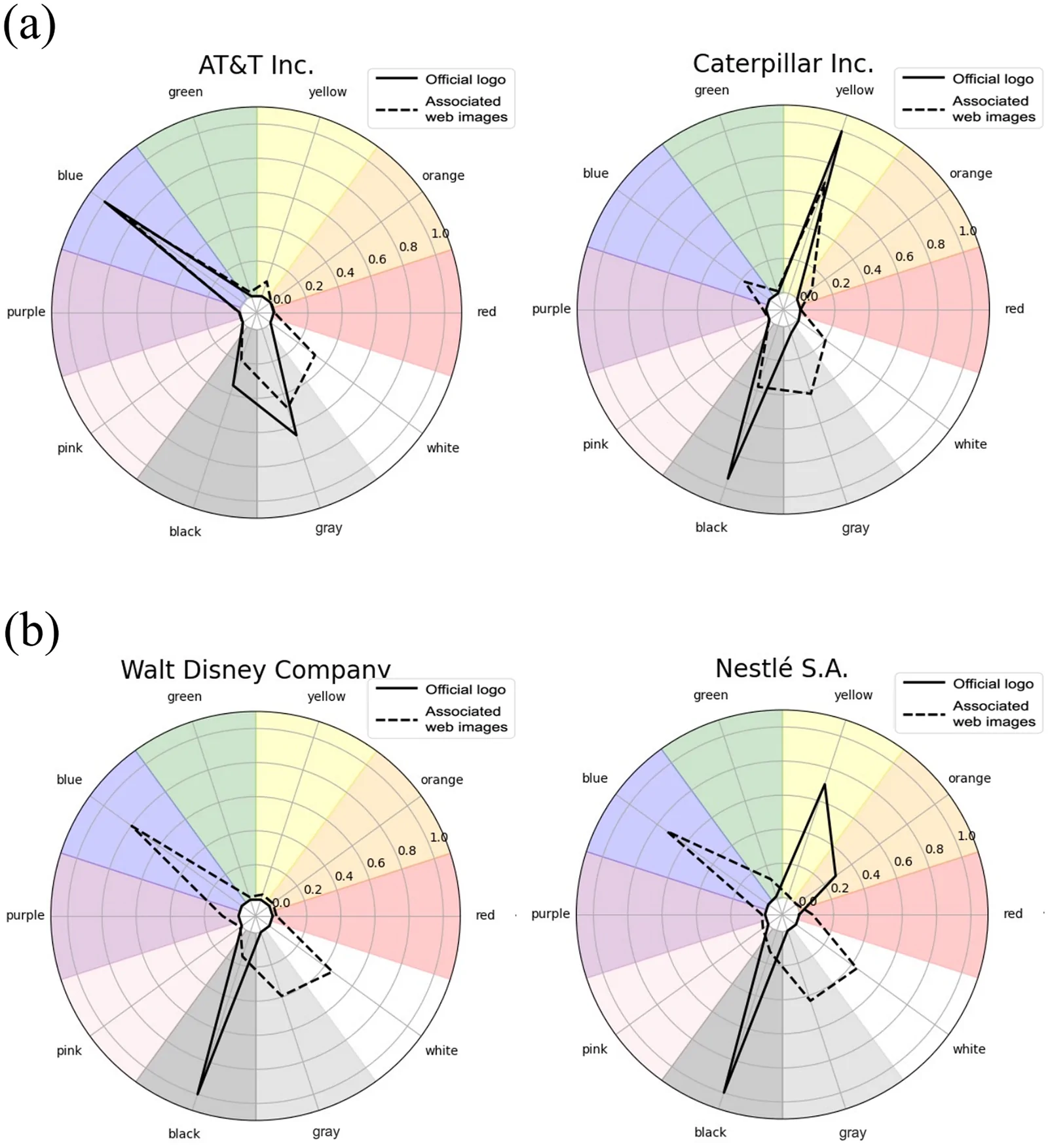
Comparison between the 10-dimensional color vectors extracted from official logos and from the screenshot of the associated web images, in four illustrative cases. Each radar shows the color vector of the logo (solid line) and that of the associated web images (dotted line). (a) For AT&T and Caterpillar, the logo and web-image vectors largely overlap, with the corporate signature colors (blue for AT&T; yellow and black for Caterpillar) dominating both. (b) For Walt Disney and Nestlé, the web-image vector substantially spreads over more base colors than the logo, reflecting the broader range of color used in their products including films, theme parks, and consumables associated with these brands.

### Color use in literature

The two 20th-century Anglo-American poets Amy Lowell and Georgia Douglas Johnson whose corpora we analyze are considered as representative of early Imagism and the Harlem Renaissance–-movements understood to reflect divergent, culturally significant aesthetic strategies–-respectively [81,82].

Imagist poets such as Lowell are said to have sought to capture sensory perception with precision and lucidity while minimizing symbolic or moral associations [83–86] whereas, in contrast, Harlem Renaissance poets such as Johnson often employed visual imagery to articulate racial and cultural identity by incorporating color into the broader discourse of modern Black expression [87,88]. These differing aesthetic directions are known to manifest differently in color use [81–83]. Using our method, we can measure and characterize the differences in color use between the writers from their corpora.

In Table 3 we show the color association values of Lowell and Johnson, the component-wide differences Δ, the *z*-score of each Δ relative to the permutation null distribution, and the two-sided permutation *p*-value. The differences are also visualized in Fig 6. In Lowell’s works we find that White, Yellow, Green, and Gray are stronger, and by contrast in Johnson’s work we find that Black and Blue are noticeably stronger, with Red, Purple, and Pink mildly so. These results show (along with Fig 6 illustrating this in a graphically intuitive manner) the differing color palettes of the poets: Lowell exhibits stronger associations with lighter colors (most prominently White and Yellow), whereas Johnson does so with darker colors (most prominently Black and Blue). This demonstrates our method’s capability of characterizing the color use of poets in a way that not only can corroborate traditional qualitative scholarship, but also achieve it with more detail and nuance thanks to its quantitative nature.

**Table 3:** Color association strengths of Lowell and Johnson, and their differences. Shown are the color vectors of the two poets, the differences Δ (Lowell – Johnson), the *z*-score of each Δ against the permutation null ($z=\Delta_{obs}/{SD}_{null}$), and the two-sided *p*-value from the same test (*B* = 10,000 size-matched partitions; see Methods). |z|≫1 indicates that Δ is significantly larger than a random partitioning would produce.

Color	Lowell	Johnson	Δ	*z* (perm)	*p* (perm)
Red	0.08736	0.09118	−0.00382	−2.0	0.049
Orange	0.14076	0.14059	−0.00017	−0.1	0.942
Yellow	0.18741	0.17590	−0.01151	−4.9	< 10^–4^
Green	0.16106	0.15158	−0.00948	−3.6	< 10^–3^
Blue	0.33984	0.35290	−0.01306	−3.5	< 10^–3^
Purple	0.04969	0.05242	−0.00273	−2.3	0.019
Pink	0.03388	0.03543	−0.00155	−1.9	0.056
Black	0.24204	0.26686	−0.02482	−10.0	< 10^–4^
Gray	0.47613	0.47051	−0.00563	−2.6	0.010
White	0.28183	0.26263	−0.01919	−6.2	< 10^–4^

**Fig 6:**
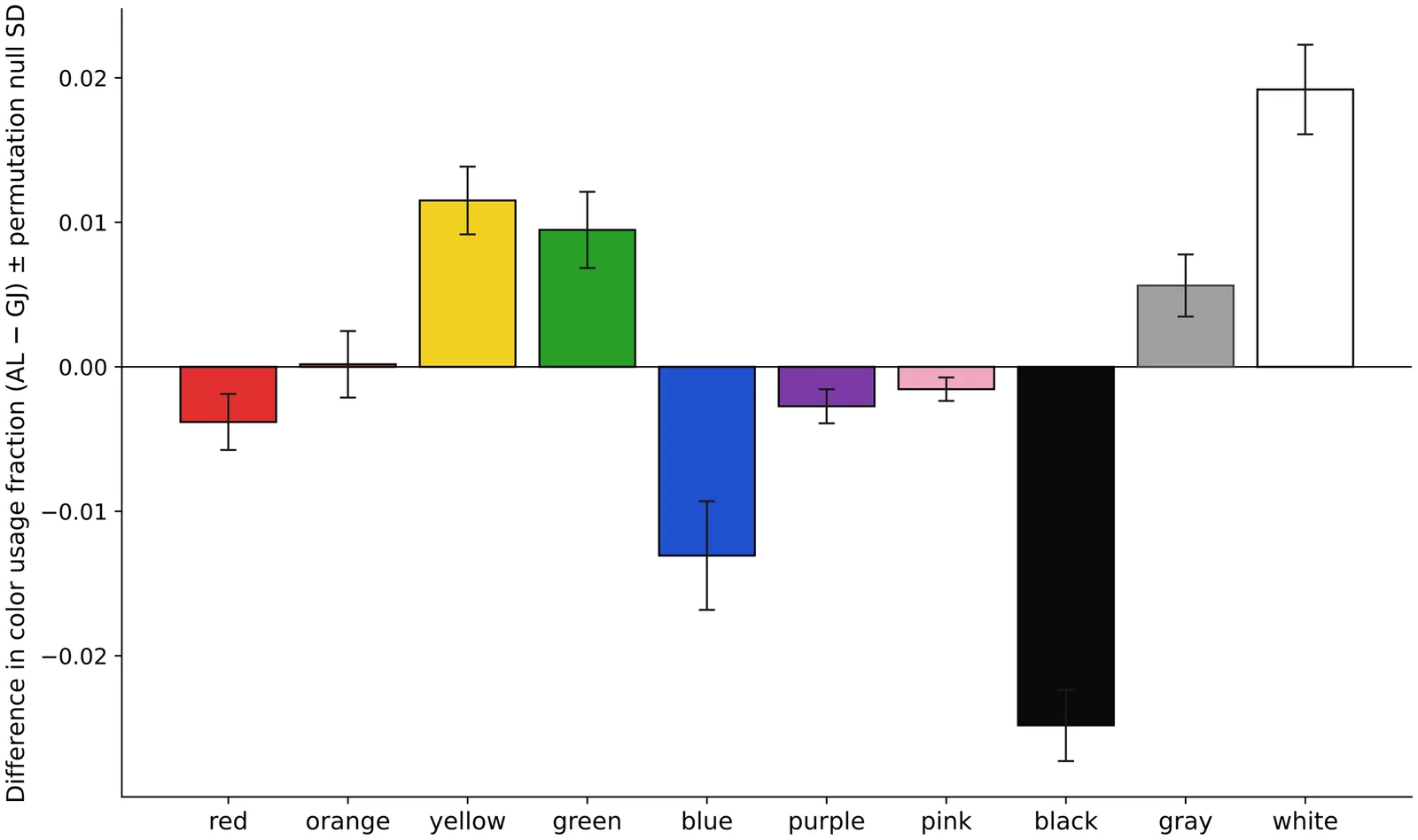
Differences in color association of the works of Amy Lowell and Georgia Douglas Johnson. Differences Δ (Lowell – Johnson) for the ten base colors. Positive bars mark those colors stronger in Lowell; negative bars, those colors stronger in Johnson. Error bars show the standard deviation of the permutation null (*B* = 10 000; see Methods).

## Conclusion

In this paper we presented a new robust and practical computational method for constructing word-color associations in the CIELch color space using publicly accessible large-scale image data for a given word. It allowed us to overcome several limitations found in traditional methods including insufficient data, potential researcher bias, and a significant loss of information originating from single-color assignments. Additionally, our method does not suffer from the serious crucial shortcomings of LLM-based methods, namely the lack of methodological transparency. We applied our method to two domains where color plays a critical role in communication, corporate branding and literature, where we were able to demonstrate its potential for providing quantitative answers to and helping us form new perspectives on long-held questions. In corporate branding, we showed that the relationships between the colors in corporations’ official logos show differing levels of correlation with various associated images depending on the industry sector. In literature, we quantified the differences between two prominent poets known for their color symbolism, the Imagist Amy Lowell and Georgia Douglas Johnson of the Harlem Renaissance, who have long been considered to exhibit starkly contrasting uses of color in their works.

Based on these findings, we anticipate more fruitful and intriguing uses of our method in discovering yet new areas or problems in word-color association. We also suggest multiple avenues for improving our method and widening the horizon of its application. First, a deeper exploration into understanding the base colors and their numerical representation would be welcome; the color brown, for instance, is commonly considered a base color across many cultures but was excluded in our framework as it posed practical difficulties since it showed a significant overlap with other chromatic colors (especially orange and yellow) and achromatic ones simultaneously. Since our method is inherently flexible regarding the selection of base colors, however, it could easily accommodate additional or alternative ones as it becomes necessary and feasible to do so. Second, a more sophisticated preprocessing technique for treating longer text segments would be helpful for the study of literary texts that contain highly elaborate sentences involving images and sentiments. For instance, while our method is capable of capturing the color implications of word groups in its current form (e.g., the combination “peeled pear” would map to the pale shade expected of a pear when processed as a single unit), a more advanced lemmatization and part-of-speech preprocessing step that does not separate the modifiers (“peeled”) from the modified words (“pear”) would enable an even deeper color inference, as would text-parsing methods that preserve long-range correlations between words. Third, an in-depth, large-scale study of general word-color association in a given language would be illuminating in both linguistic and cultural terms. Since our framework is fundamentally language-agnostic by design and web image search engines are readily available in many languages, one could perform a common set of analyses across multiple languages for intriguing opportunities for cross-linguistic and cross-cultural investigation. Fourth, the framework can be extended to studying the temporal dynamics of word-color associations, a promising direction given recent computational findings that have used contemporary embeddings to quantify long-term historical changes in language [89,90]. There are works that document the common features of fundamental color terms, partially preserved across cultures and periods [4,19,58,91,92]. Many specific color associations are also historically dynamic, including shifts in the cultural meanings of individual colors over decades such as the twentieth-century gendering of pink, and even centuries [93–97]. Google image search’s date-range filter that restricts queries to images indexed within a specified period would be helpful here as it enables a direct comparison of color vectors for the same query across many historical time windows.

## Supporting information

S1 FigStability of the color vector across the depth of the search results.For each of 30 randomly sampled common nouns, the results were collected down to ten page-scrolls, giving one viewport color vector per page (28 of the 30 words reached page 10; the other two stopped at page 9 and page 8). (a) Mean cosine between the color vectors of every pair of pages (*N* = 30 words). The overall mean is ≥0.94; the mean declines only slowly with page distance, from 0.962 at distance 1 to 0.947 at distance 9 (Pearson r=−0.98, $p=2.2\times 10^{-6}$). (b) For each word, *K* pages were drawn at random from pages 2–10 and their color vectors averaged (100 draws per word and *K*); boxplots show the cosine between this average and the top viewport (page 1). The median is ≥0.97 for every *K*.(PDF)

S2 Fig Polysemy sensitivity on a random sample of 30 common English nouns. For each word *w* we automatically construct one bare (no sense disambiguation) query and one sense-disambiguated query from WordNet noun synset; the added sense word is selected from the synset’s lemma_names by a fixed rule (after removing the query word itself, preference is given to single-word over compound, then shortest, then alphabetical). (a) Within-word color-vector spread (maximum pairwise cosine distance among the sense-specific 10-dimensional color vectors) plotted against WordNet noun sense count *K*(*w*); points sharing the same integer *K* are horizontally jittered for label readability. (b) Representative high-spread words: each row is the 10-dimensional named base-color ratio profile (Red, Orange, Yellow, Green, Blue, Purple, Pink, Black, Gray, and White) for the bare query (top row) and for each sense-disambiguated query (subsequent rows, labeled with the added sense word); each row is normalized to sum to 1 across the ten base colors.
(PDF)

S3 FigDistribution of pure white pixels in screenshots.Histogram of the fraction of pure white pixels from 100 randomly chosen screenshots used to estimate the fraction of the white dividers between images. Based on this analysis, we eliminate the average fraction, 31.42%, of the brightest (highest lightness *L*) pixels. The Kolmogorov–Smirnov (KS) test does not reject normality for the number of white pixels (*p* = 0.1675). The mean 0.3142 and the standard deviation 0.0114 are color-indicated: black (mean), blue (±1 SD), and light blue (±2 SD).(PDF)

S4 Fig Obtaining the color coordinate distributions of the chromatic and the achromatic base colors. (a) The hue *h* distribution for Blue obtained by combining the *h* values from images returned for the color synonyms according to the Oxford American Writer’s Thesaurus [61,62]. (b) Lightness *L* distribution for White similarly obtained by combining the *L* values from the images returned for the color synonyms. The detailed distributions for the colors and synonyms are given in S5 Fig.
(PDF)

S5 Fig Frequency distributions of color synonyms. The figures are categorized into two types: the hue (*h*) distributions and the lightness (*L*) distributions. The hue distributions represent the distribution of hues for synonyms of chromatic terms, including Red, Orange, Yellow, Green, Blue, Purple, and Pink. These figures visualize the hue variations associated with each color synonym, providing insights into the chromatic diversity of these colors. The lightness distributions display the distribution of lightness values for synonyms of achromatic terms, including Black, Gray, and White. These figures illustrate how achromatic colors vary in brightness, offering a detailed look into the gradations of these colors. For all figures, the *y*-axis represents frequency with a fixed range from 0 to 1500. The *x*-axis ranges from 0 to 360 for hue *h* and from 0 to 100 for lightness *L*.
(PDF)

S6 Fig Chroma distributions of representative achromatic terms. The chroma distributions of 15 representative achromatic terms listed in Table 1 are visualized. The histograms display the frequency of chroma values for each term, with the red dashed line indicating the chroma threshold for each term and the green solid line representing the maximum threshold (15.93) across all terms. Over 95% of pixels for each term fall within the specified range of chroma.
(PDF)

S7 Fig Validation against the NRC Word-Colour Association Lexicon on a random 100-word sample. Words carrying both an NRC label and a framework vector were sampled at random. The lexicon gives one label per word and the framework a distribution, so the agreement is measured by ranking and not an exact match. (a) Per-color one-vs-rest AUC: for each base color, its ratio ranks the words whose NRC label is that color above the rest, with the dashed line at 0.5 marking chance. (b) Confusion matrix between the NRC-assigned color (rows) and the framework’s argmax color (columns), restricted to the ten base colors shared by both schemes; values are raw fractions. Brown, absent from the base-color set, does not label any sampled word.
(PDF)

S8 Fig Per-concept comparison against the human ratings of Rathore et al. (2019). Each palette color, given as a CIELch coordinate, is mapped to one base bin by the chromatic-threshold rule in the manuscript–-the chromatic colors to their ranges of hue, the achromatic colors to their ranges of lightness. The ratings are then summed per concept within each bin and the chromatic and the achromatic blocks are normalized separately. Nine of the 18 concepts reuse the existing per-word vectors; the other nine were re-scraped, disambiguating the query when the bare term is polysemous. For each concept (12 fruits left of the dashed line; 6 materials right), the cosine similarity between the framework’s prediction and the aggregated human ratings is shown for three slices: the full 10-d vector (blue), the chromatic block alone (red), and the achromatic block alone (gray).
(PDF)

S9 FigThe Setlur & Stone (2016) method alignment against the present framework on 100 random English words.Per-color one-vs-rest AUC of the framework’s per-color ratio in ranking Setlur full-pipeline color-*c* labels above non-*c*. Sample: 94 evaluable words (6 of 100 returned no images and are excluded). Per-color counts of Setlur positives are annotated above each bar; the median over defined colors (blue and pink have no Setlur positives in this sample, hence undefined) is 0.63. Top-1 agreement: 31/94≈33%.(PDF)

S10 FigRepresentational Similarity Analysis (RSA) between the framework and comp-syn on random 100-word samples.The two embeddings partition color space differently (ten named CIELch bins versus eight JzAzBz octants), so their dimensions do not overlap, and are compared via global similarity structure rather than dimension by dimension. (a) Pairwise-cosine scatter on a representative draw (the draw whose RSA Pearson *r* is closest to the median across the 100 random samples of 100 words each; 4 950 word pairs per draw), with the framework’s 10-d CIELch space on the *x*-axis and comp-syn’s 8-d JzAzBz cube histogram on the *y*-axis; the gray dashed line marks identity. The representative draw yields RSA Pearson *r* = 0.458 and Spearman ρ=0.425. (b) Distribution of RSA Pearson *r* across the 100 random draws (median 0.458, range [0.334, 0.618]), overlaid with the row-shuffled null distribution computed on the representative draw ($r_{null,max}=0.119$); all 100/100 observed draws lie above the null maximum.(PDF)

S11 FigRobustness of the per-color Lowell–Johnson contrast to the relative weight of indirect color-associated terms.Per-color $\Delta =f_{c}^{\text{Lowell}}-f_{c}^{\text{Johnson}}$ plotted against the indirect-word weight α∈[0.1,1.0] in steps of 0.1. Error bars are bootstrap standard errors; each base color is plotted in its corresponding color. The α=0 extreme is excluded because Johnson’s corpus contains only 22 direct-color tokens.(PDF)

S12 FigPermutation null distributions for the per-color Lowell–Johnson contrasts.For each base color, the pooled Lowell–Johnson content-word lexicon is partitioned at random into two size-matched corpora (*B* = 10,000); the resulting per-color Δ values form the null histogram. The vertical red line marks the observed Δ under the actual partition; each panel reports the corresponding *z*-score and empirical *p*-value.(PDF)

S1 Table Methodological comparison of image- and lexicon-based word-color frameworks. (PDF)

S2 Table Data Sources for Lowell’s and Johnson’s Poetry. (PDF)

S3 Table Brands grouped by industry sector. (PDF)

S4 Table Cosine similarity between official logos and associated web images for the top 100 global companies. (PDF)

S1 File Software module for word-color association. The module (provided as S1_File.zip) contains the complete research code and underlying data required to replicate the findings of this study. It builds word-color associations by retrieving images linked to search terms, extracting their colors, and representing the words in terms of color composition. In addition to quantifying color information, the module provides diverse visualizations of the results. It also includes specialized scripts and datasets for analyzing visual elements of brand names and literary text corpora. Comprehensive documentation and usage instructions are provided in README.md.
(ZIP)
