# Supplementary material for: Word-color association from large-scale online data: Methodology and applications
Source: PLoS One. 2026 Oct 16;21(10):e0360407. doi: 10.1371/journal.pone.0360407 (PMC13674752; doi:10.1371/journal.pone.0360407)
Supplement: Supplementary file 1 [file pone.0360407.s001.pdf]

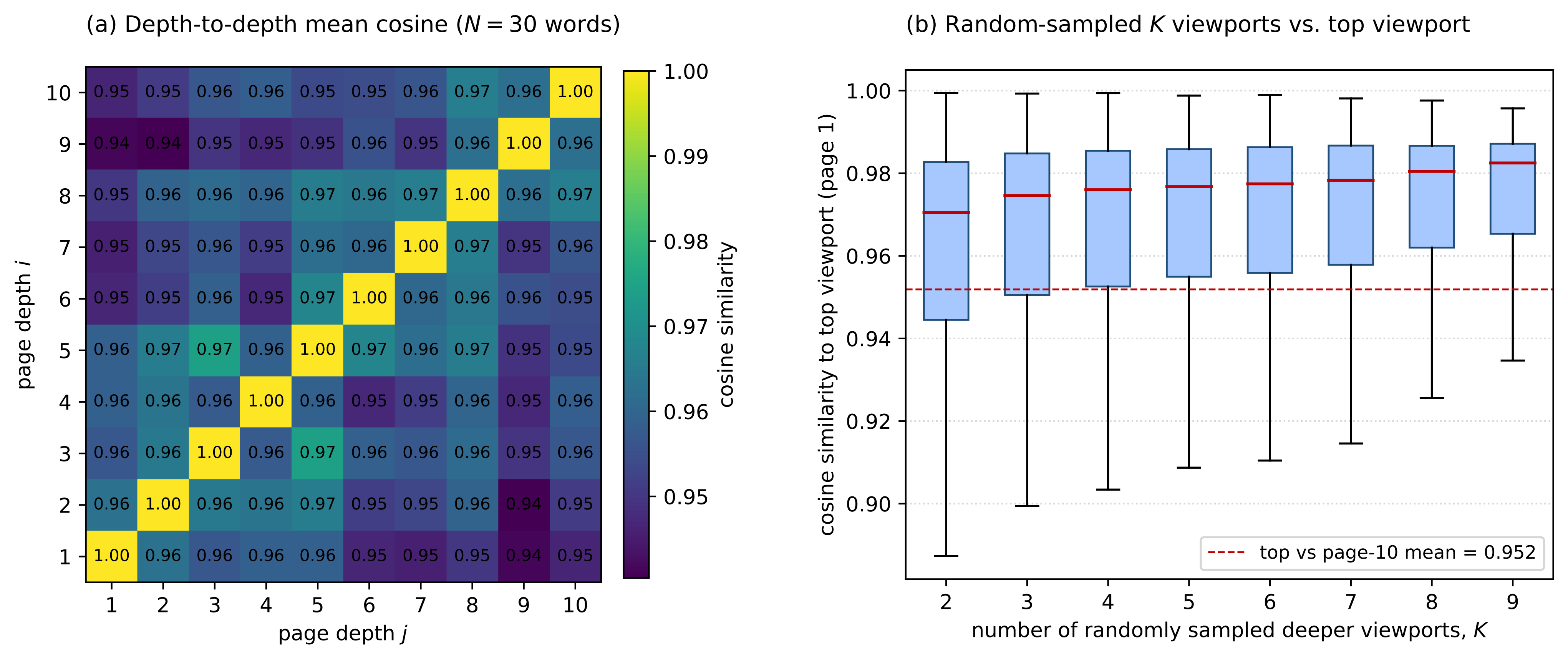

(a) Depth-to-depth mean cosine (N = 30 words)
page depth i
page depth j
cosine similarity
(b) Random-sampled K viewports vs. top viewport
cosine similarity to top viewport (page 1)
number of randomly sampled deeper viewports, K
top vs page-10 mean = 0.952
