# Supplementary material for: Word-color association from large-scale online data: Methodology and applications
Source: PLoS One. 2026 Oct 16;21(10):e0360407. doi: 10.1371/journal.pone.0360407 (PMC13674752; doi:10.1371/journal.pone.0360407)
Supplement: Supplementary file 2 [file pone.0360407.s002.pdf]

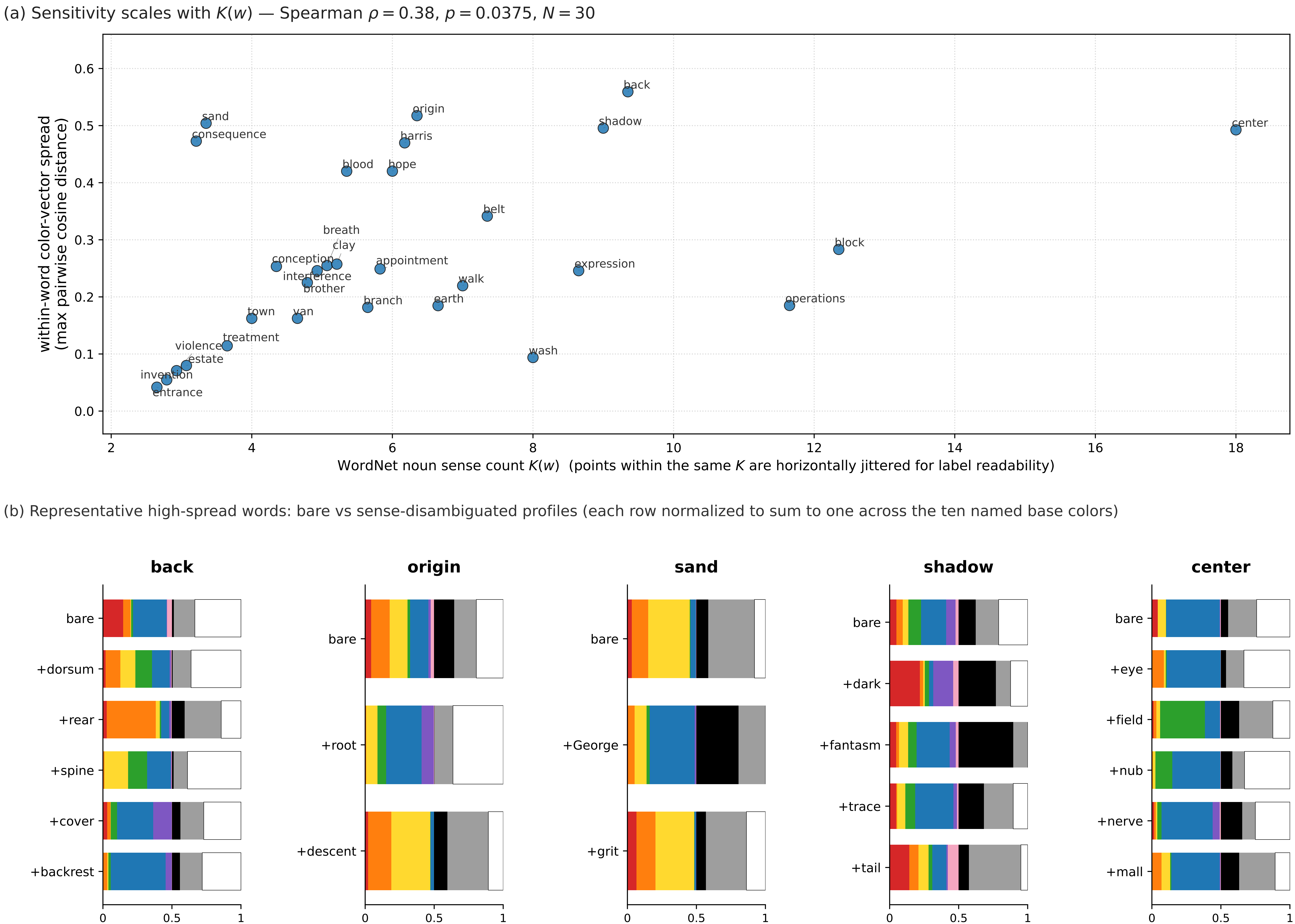

(a) Sensitivity scales with K(w) — Spearman ρ = 0.38, p = 0.0375, N = 30
within-word color-vector spread (max pairwise cosine distance)
WordNet noun sense count K(w) (points within the same K are horizontally jittered for label readability)
back
origin
shadow
center
sand
consequence
barris
blood
hope
belt
breath
clay
conception
interference
brother
appointment
walk
expression
block
operations
town
van
branch
earth
treatment
violence
estate
invention
entrance
wash
(b) Representative high-spread words: bare vs sense-disambiguated profiles (each row normalized to sum to one across the ten named base colors)
back
bare
+dorsum
+rear
+spine
+cover
+backrest
origin
+root
+descent
sand
+George
+grit
shadow
+dark
+fantasm
+trace
+tail
center
+eye
+field
+nub
+nerve
+mall
