# Supplementary material for: Word-color association from large-scale online data: Methodology and applications
Source: PLoS One. 2026 Oct 16;21(10):e0360407. doi: 10.1371/journal.pone.0360407 (PMC13674752; doi:10.1371/journal.pone.0360407)
Supplement: Supplementary file 3 [file pone.0360407.s003.pdf]

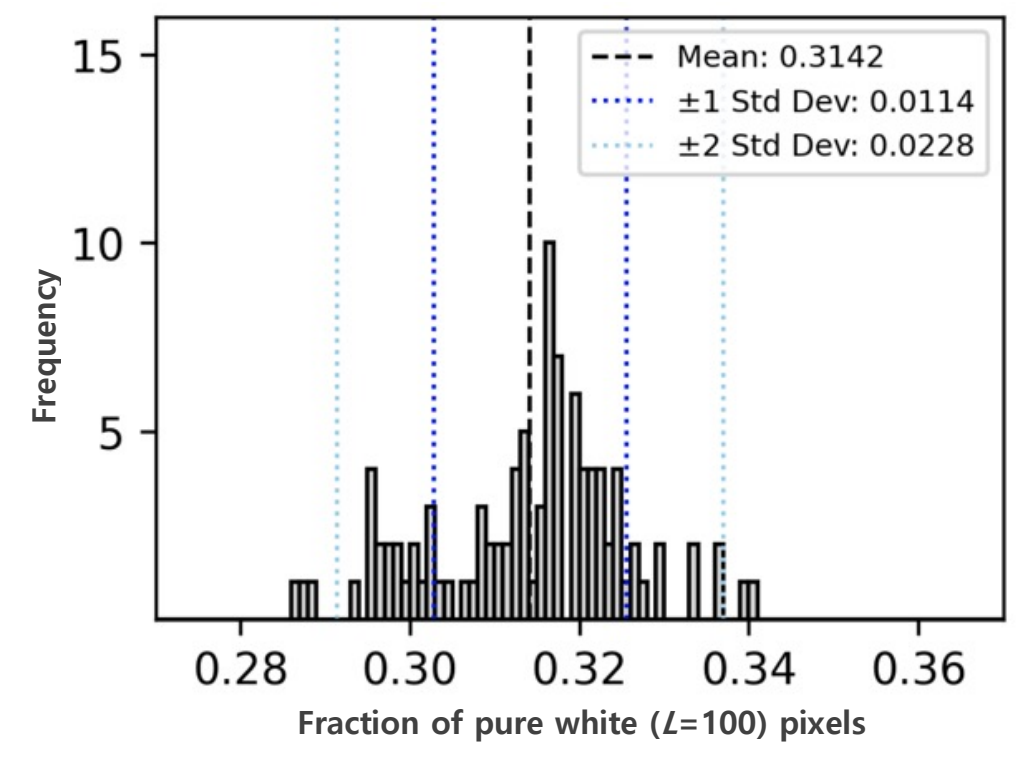

Mean: 0.3142
±1 Std Dev: 0.0114
±2 Std Dev: 0.0228
15
10
5
Frequency
0.28
0.30
0.32
0.34
0.36
Fraction of pure white (L=100) pixels
