# Supplementary material for: Word-color association from large-scale online data: Methodology and applications
Source: PLoS One. 2026 Oct 16;21(10):e0360407. doi: 10.1371/journal.pone.0360407 (PMC13674752; doi:10.1371/journal.pone.0360407)
Supplement: Supplementary file 4 [file pone.0360407.s004.pdf]

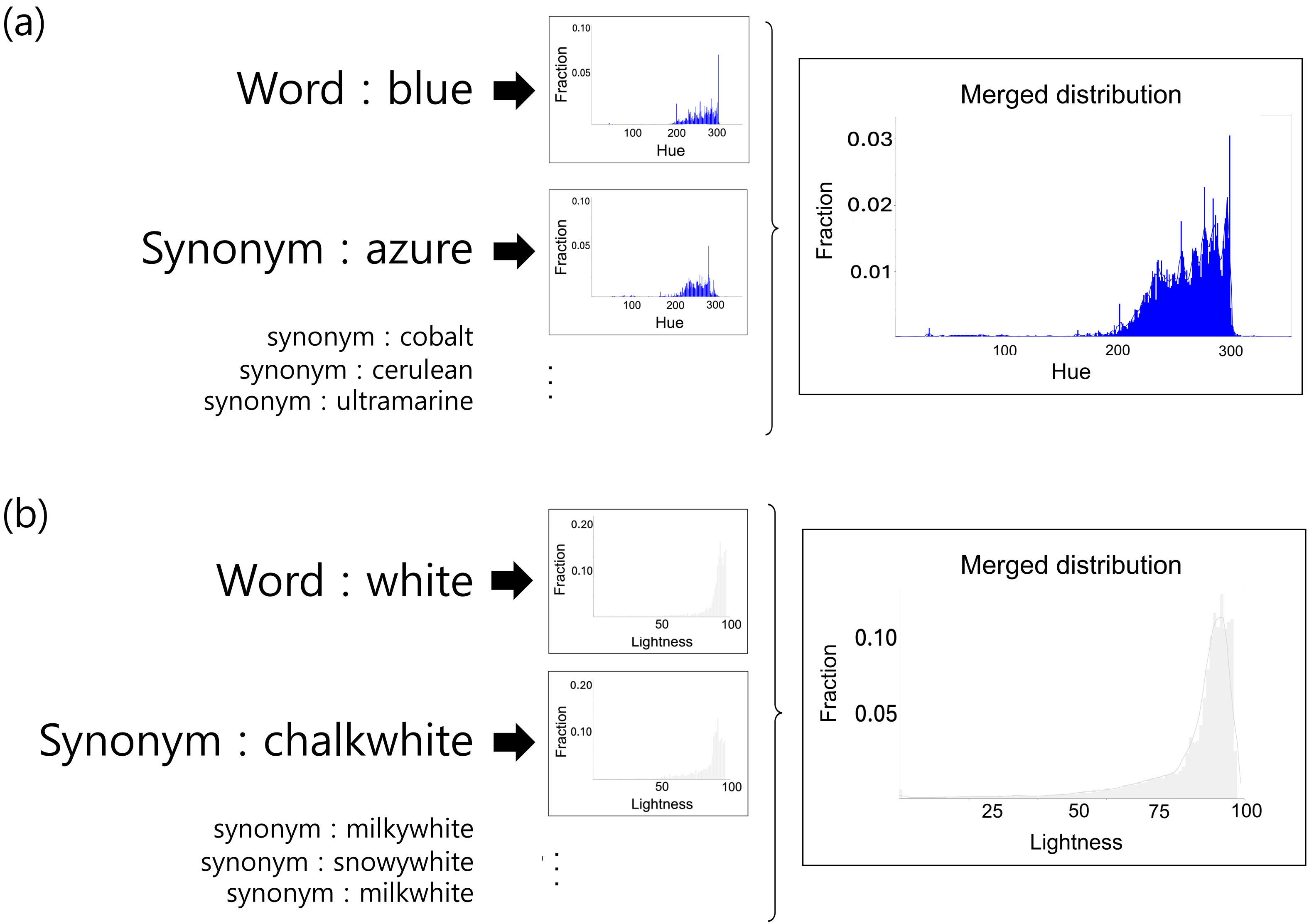
(a)
Word : blue
Synonym : azure
synonym : cobalt
synonym : cerulean
synonym : ultramarine
Fraction
Hue
0.10
0.05
100
200
300
Merged distribution
0.03
0.02
0.01
(b)
Word : white
Synonym : chalkwhite
synonym : milkywhite
synonym : snowywhite
synonym : milkwhite
Lightness
0.20
50
25
75
