# Supplementary material for: Word-color association from large-scale online data: Methodology and applications
Source: PLoS One. 2026 Oct 16;21(10):e0360407. doi: 10.1371/journal.pone.0360407 (PMC13674752; doi:10.1371/journal.pone.0360407)
Supplement: Supplementary file 5 [file pone.0360407.s005.pdf]

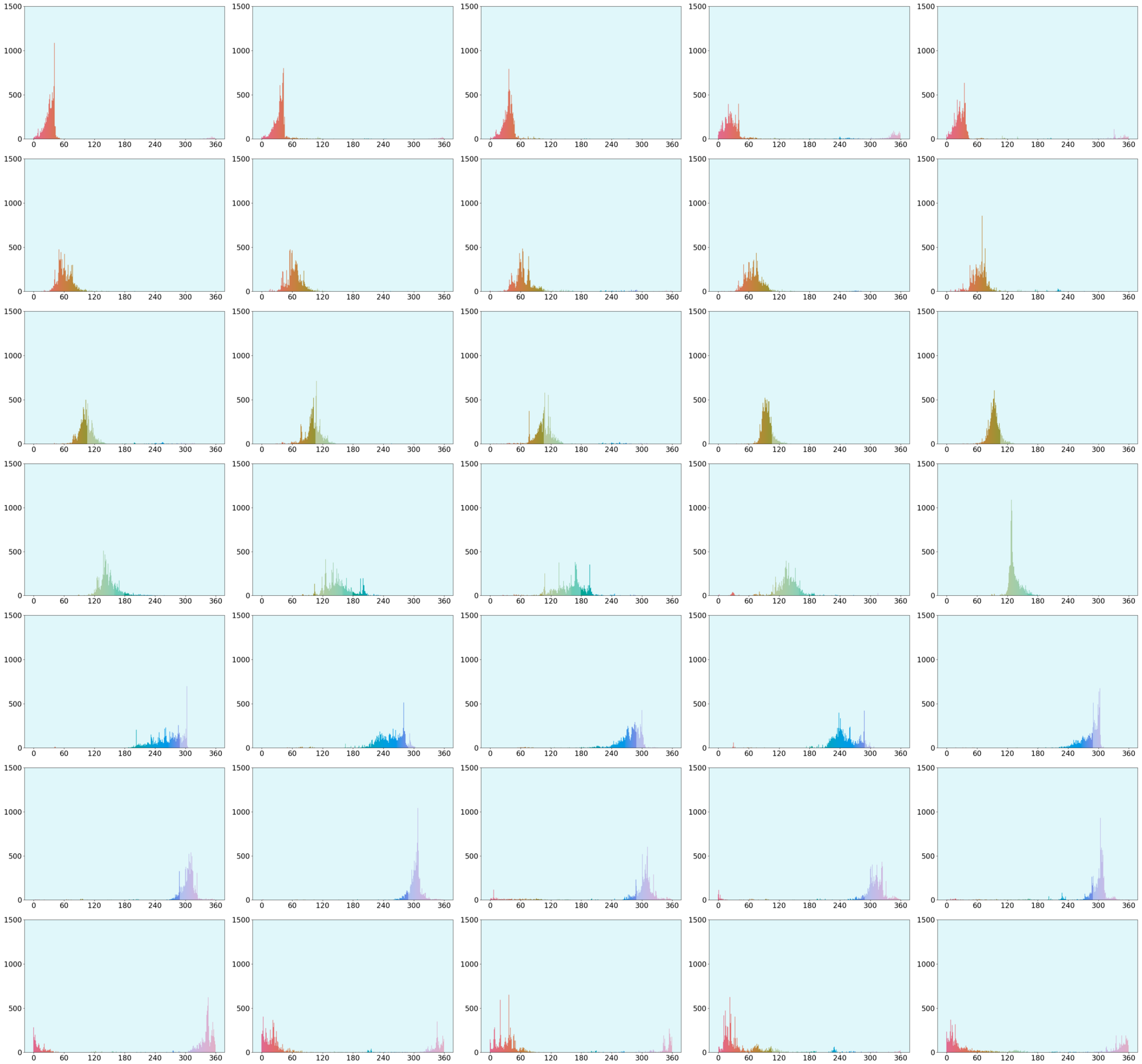

1500
1000
500
0
0 60 120 180 240 300 360

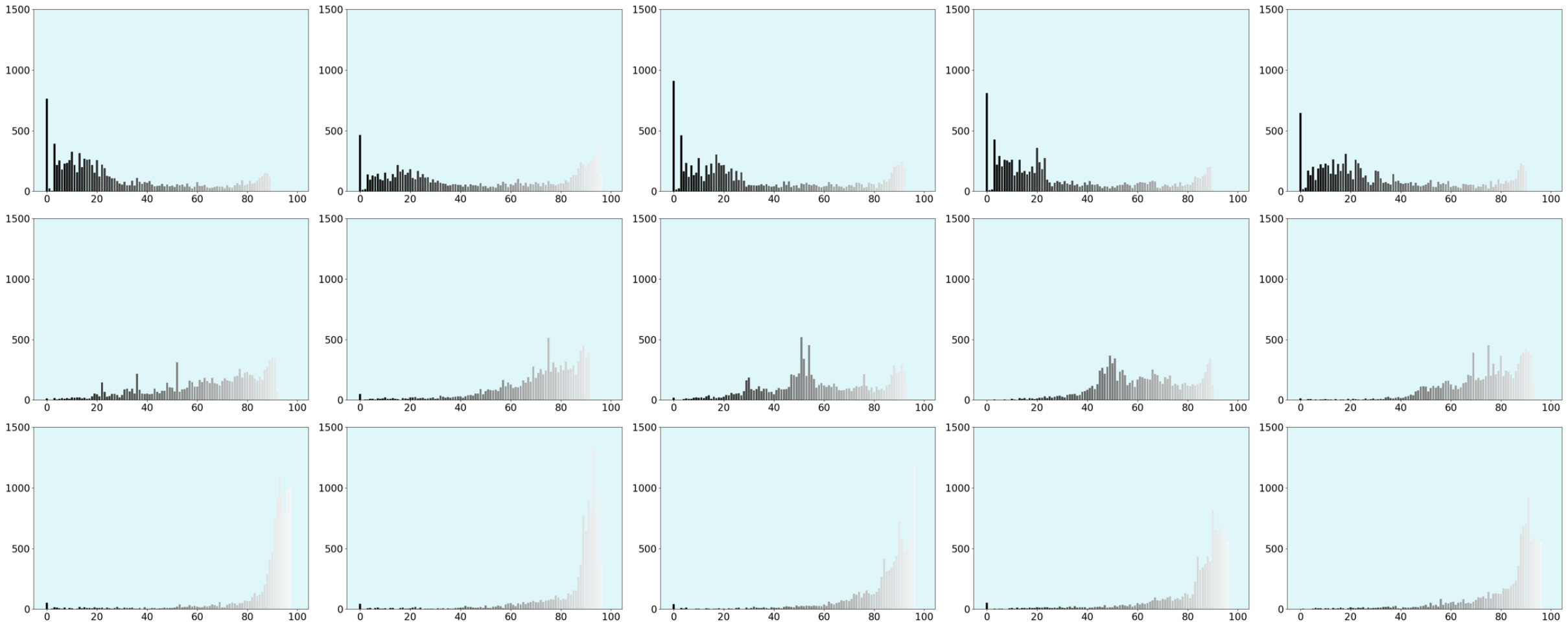

1500
1000
500
0
0 20 40 60 80 100
