# Supplementary material for: Word-color association from large-scale online data: Methodology and applications
Source: PLoS One. 2026 Oct 16;21(10):e0360407. doi: 10.1371/journal.pone.0360407 (PMC13674752; doi:10.1371/journal.pone.0360407)
Supplement: Supplementary file 6 [file pone.0360407.s006.pdf]

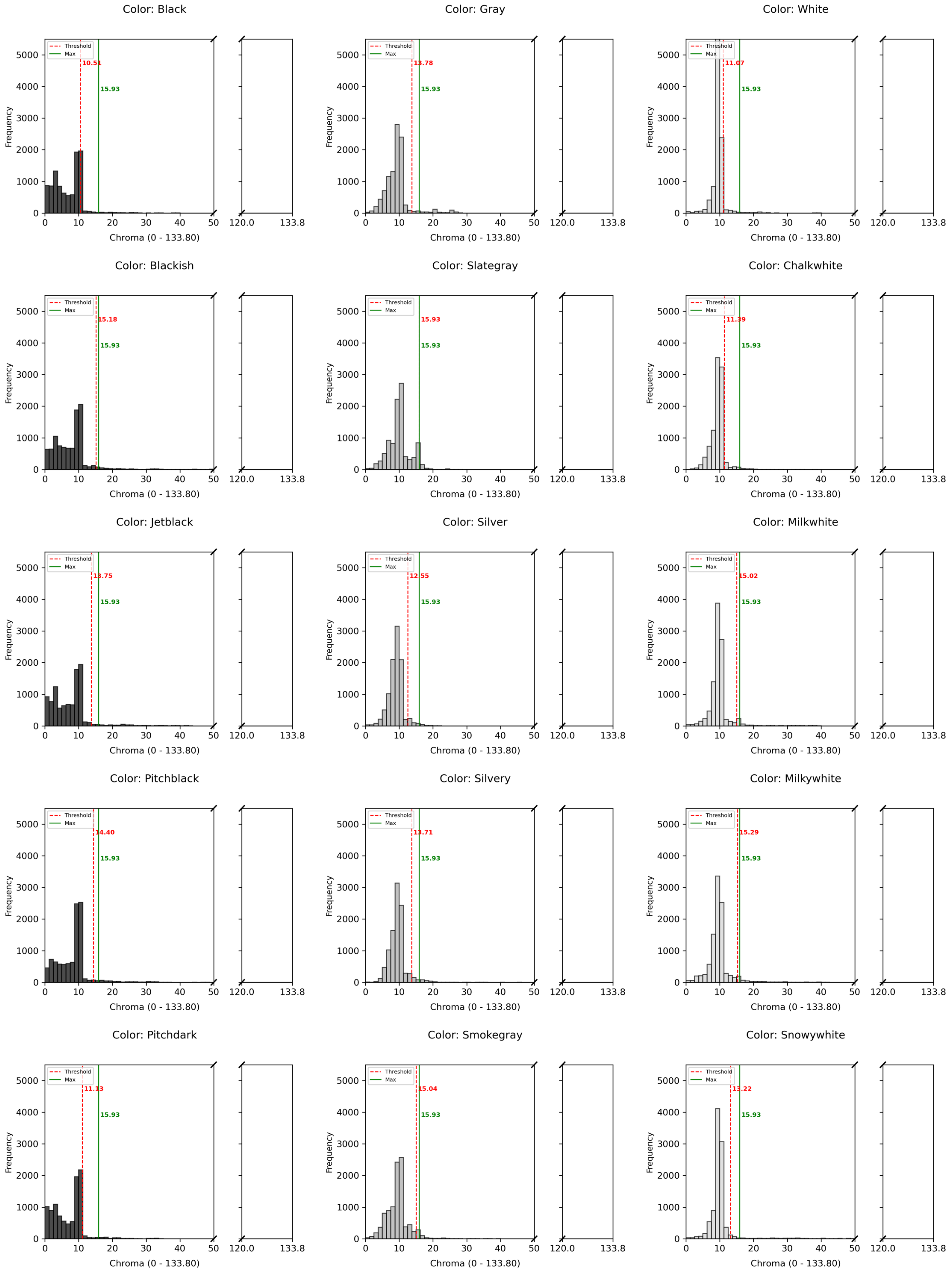
Color: Black
Threshold
Max
10.51
15.93
Color: Gray
Threshold
Max
13.78
15.93
Color: White
Threshold
Max
11.07
15.93
Color: Blackish
Threshold
Max
15.18
15.93
Color: Slategray
Threshold
Max
15.93
15.93
Color: Chalkwhite
Threshold
Max
11.39
15.93
Color: Jetblack
Threshold
Max
13.75
15.93
Color: Silver
Threshold
Max
12.55
15.93
Color: Milkwhite
Threshold
Max
15.02
15.93
Color: Pitchblack
Threshold
Max
14.40
15.93
Color: Silvery
Threshold
Max
13.71
15.93
Color: Milkywhite
Threshold
Max
15.29
15.93
Color: Pitchdark
Threshold
Max
11.13
15.93
Color: Smokegray
Threshold
Max
15.04
15.93
Color: Snowywhite
Threshold
Max
13.22
15.93
Frequency
Chroma (0 - 133.80)
0
1000
2000
3000
4000
5000
0
10
20
30
40
50
120.0
133.8
