# Supplementary material for: Word-color association from large-scale online data: Methodology and applications
Source: PLoS One. 2026 Oct 16;21(10):e0360407. doi: 10.1371/journal.pone.0360407 (PMC13674752; doi:10.1371/journal.pone.0360407)
Supplement: Supplementary file 7 [file pone.0360407.s007.pdf]

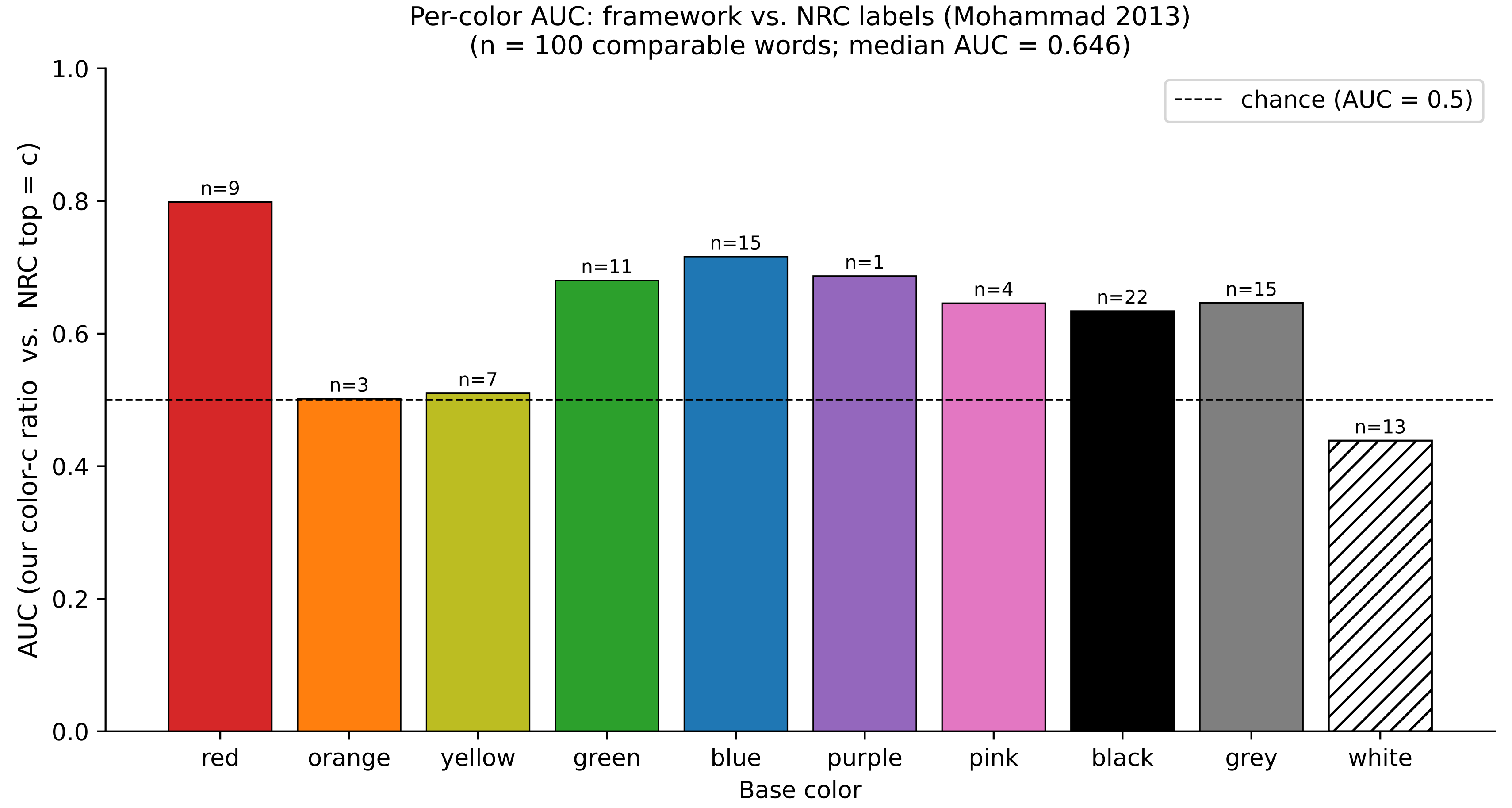

Per-color AUC: framework vs. NRC labels (Mohammad 2013)
(n = 100 comparable words; median AUC = 0.646)
AUC (our color-c ratio vs. NRC top = c)
chance (AUC = 0.5)
n=9
n=3
n=7
n=11
n=15
n=1
n=4
n=22
n=15
n=13
1.0
0.8
0.6
0.4
0.2
0.0
red
orange
yellow
green
blue
purple
pink
black
grey
white
Base color
