# Supplementary material for: Word-color association from large-scale online data: Methodology and applications
Source: PLoS One. 2026 Oct 16;21(10):e0360407. doi: 10.1371/journal.pone.0360407 (PMC13674752; doi:10.1371/journal.pone.0360407)
Supplement: Supplementary file 8 [file pone.0360407.s008.pdf]

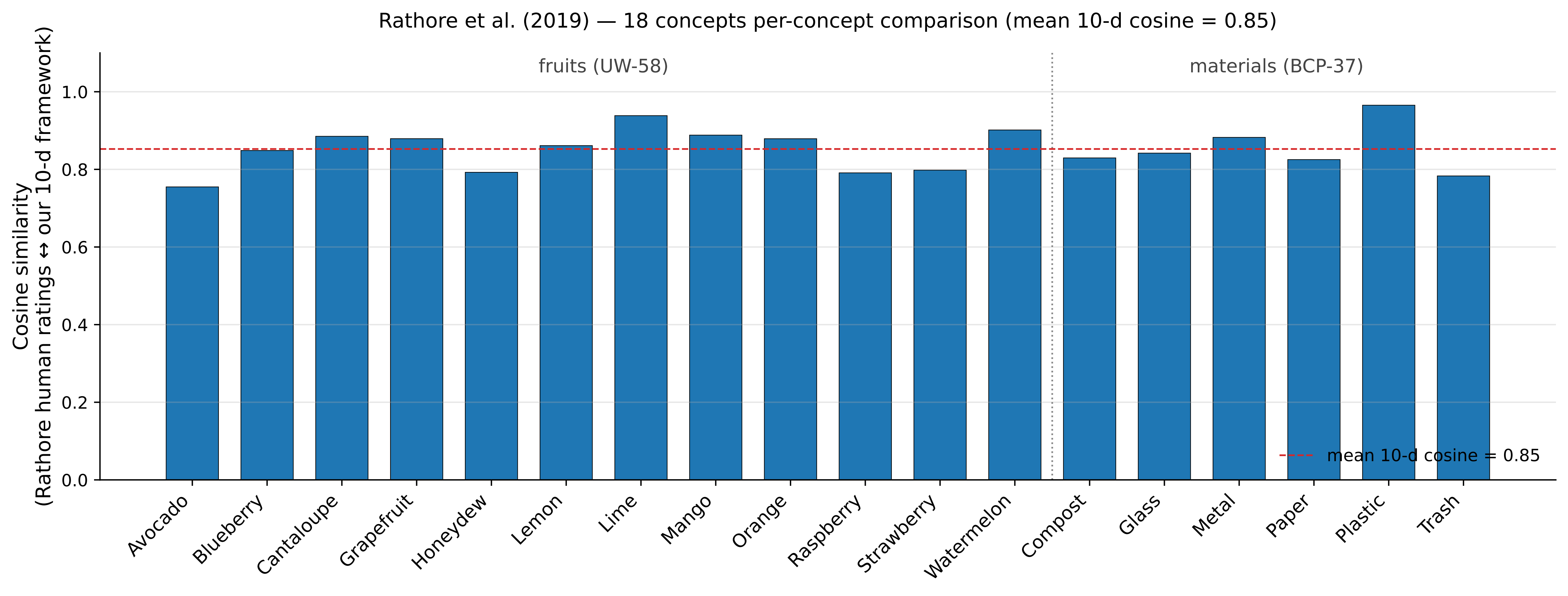

Rathore et al. (2019) — 18 concepts per-concept comparison (mean 10-d cosine = 0.85)
Cosine similarity
(Rathore human ratings ↔ our 10-d framework)
fruits (UW-58)
materials (BCP-37)
mean 10-d cosine = 0.85
1.0
0.8
0.6
0.4
0.2
0.0
Avocado
Blueberry
Cantaloupe
Grapefruit
Honeydew
Lemon
Lime
Mango
Orange
Raspberry
Strawberry
Watermelon
Compost
Glass
Metal
Paper
Plastic
Trash
