# Supplementary material for: Word-color association from large-scale online data: Methodology and applications
Source: PLoS One. 2026 Oct 16;21(10):e0360407. doi: 10.1371/journal.pone.0360407 (PMC13674752; doi:10.1371/journal.pone.0360407)
Supplement: Supplementary file 9 [file pone.0360407.s009.pdf]

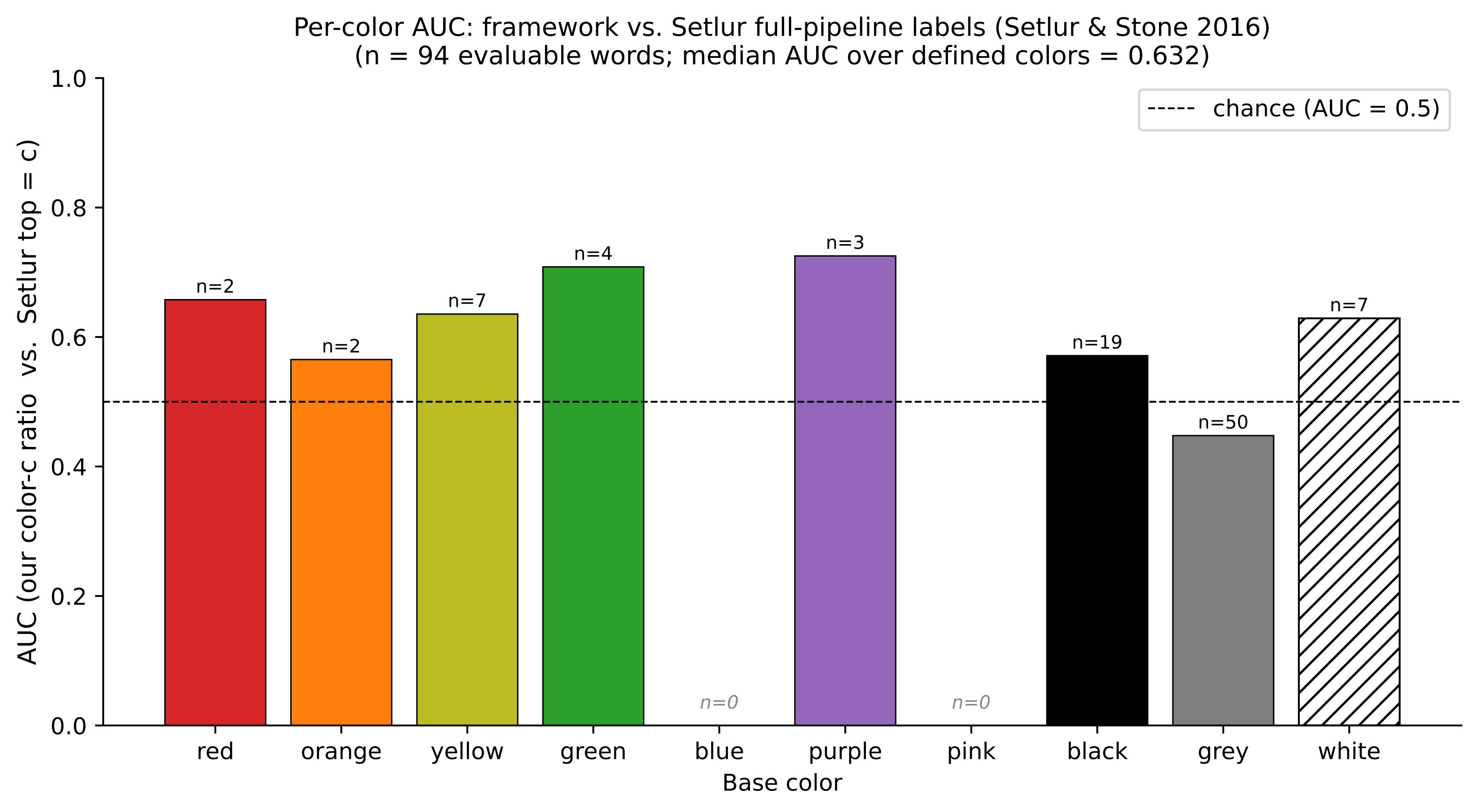

Per-color AUC: framework vs. Setlur full-pipeline labels (Setlur & Stone 2016)
(n = 94 evaluable words; median AUC over defined colors = 0.632)
chance (AUC = 0.5)
AUC (our color-c ratio vs. Setlur top = c)
1.0
0.8
0.6
0.4
0.2
0.0
n=2
n=2
n=7
n=4
n=0
n=3
n=0
n=19
n=50
n=7
red
orange
yellow
green
blue
purple
pink
black
grey
white
Base color
