# Supplementary material for: Word-color association from large-scale online data: Methodology and applications
Source: PLoS One. 2026 Oct 16;21(10):e0360407. doi: 10.1371/journal.pone.0360407 (PMC13674752; doi:10.1371/journal.pone.0360407)
Supplement: Supplementary file 10 [file pone.0360407.s010.pdf]

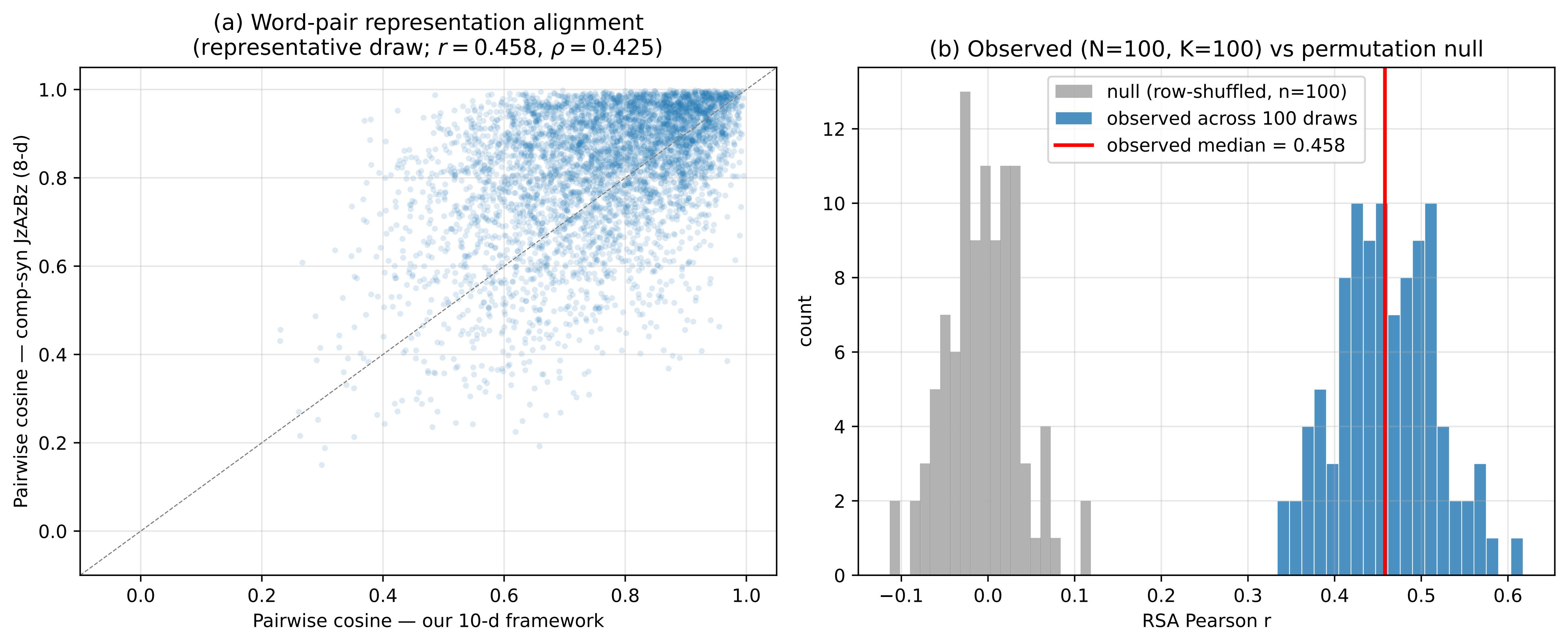
(a) Word-pair representation alignment
(representative draw; $r = 0.458$, $\rho = 0.425$)
Pairwise cosine — comp-syn JzAzBz (8-d)
Pairwise cosine — our 10-d framework
0.0
0.2
0.4
0.6
0.8
1.0
(b) Observed (N=100, K=100) vs permutation null
null (row-shuffled, n=100)
observed across 100 draws
observed median = 0.458
count
RSA Pearson r
−0.1
0.0
0.1
0.2
0.3
0.4
0.5
0.6
0
2
4
6
8
10
12
