# Supplementary material for: Word-color association from large-scale online data: Methodology and applications
Source: PLoS One. 2026 Oct 16;21(10):e0360407. doi: 10.1371/journal.pone.0360407 (PMC13674752; doi:10.1371/journal.pone.0360407)
Supplement: Supplementary file 11 [file pone.0360407.s011.pdf]

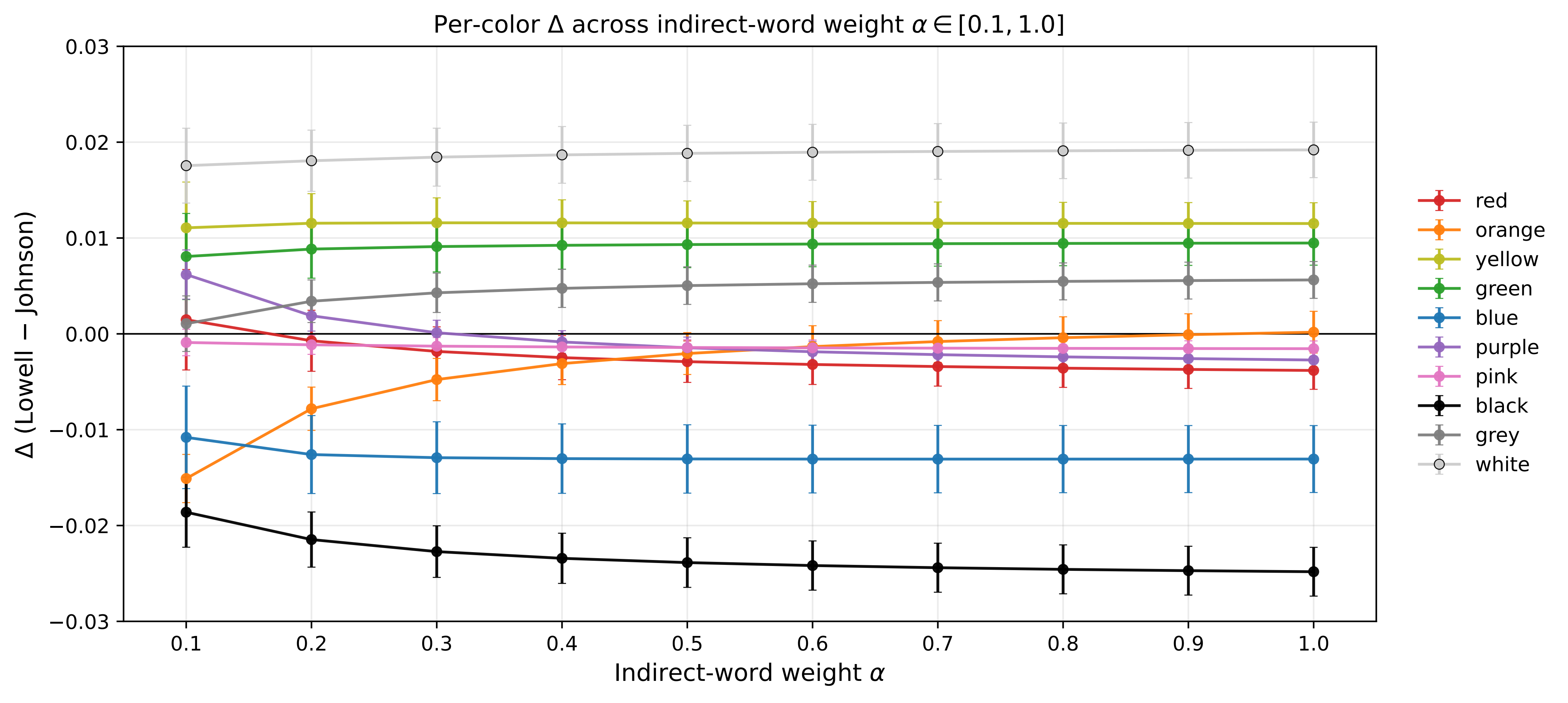

Per-color Δ across indirect-word weight $\alpha \in [0.1, 1.0]$
Δ (Lowell − Johnson)
Indirect-word weight α
0.03
0.02
0.01
0.00
−0.01
−0.02
−0.03
0.1
0.2
0.3
0.4
0.5
0.6
0.7
0.8
0.9
1.0
red
orange
yellow
green
blue
purple
pink
black
grey
white
