# Supplementary material for: Word-color association from large-scale online data: Methodology and applications
Source: PLoS One. 2026 Oct 16;21(10):e0360407. doi: 10.1371/journal.pone.0360407 (PMC13674752; doi:10.1371/journal.pone.0360407)
Supplement: Supplementary file 12 [file pone.0360407.s012.pdf]

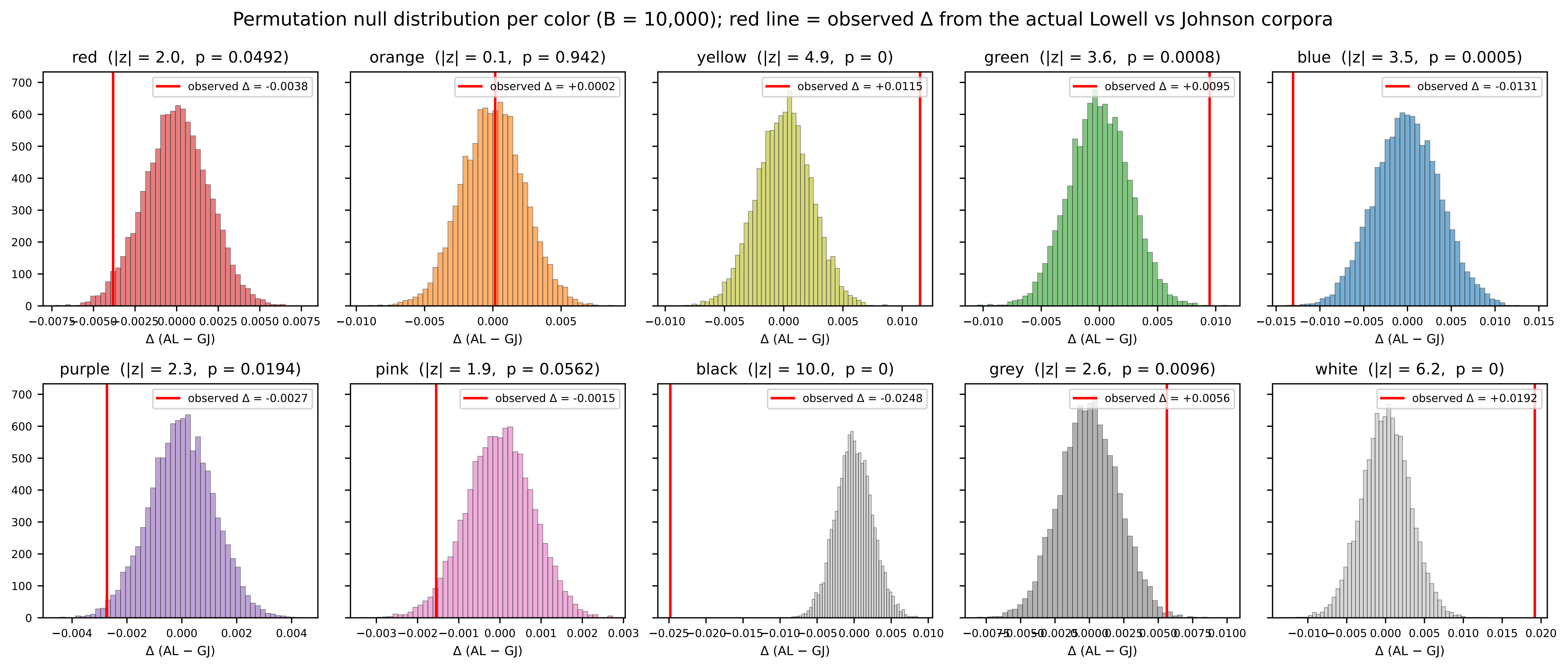
Permutation null distribution per color (B = 10,000); red line = observed Δ from the actual Lowell vs Johnson corpora
red (|z| = 2.0, p = 0.0492)
observed Δ = -0.0038
orange (|z| = 0.1, p = 0.942)
observed Δ = +0.0002
yellow (|z| = 4.9, p = 0)
observed Δ = +0.0115
green (|z| = 3.6, p = 0.0008)
observed Δ = +0.0095
blue (|z| = 3.5, p = 0.0005)
observed Δ = -0.0131
purple (|z| = 2.3, p = 0.0194)
observed Δ = -0.0027
pink (|z| = 1.9, p = 0.0562)
observed Δ = -0.0015
black (|z| = 10.0, p = 0)
observed Δ = -0.0248
grey (|z| = 2.6, p = 0.0096)
observed Δ = +0.0056
white (|z| = 6.2, p = 0)
observed Δ = +0.0192
Δ (AL − GJ)
