# Supplementary material for: Word-color association from large-scale online data: Methodology and applications
Source: PLoS One. 2026 Oct 16;21(10):e0360407. doi: 10.1371/journal.pone.0360407 (PMC13674752; doi:10.1371/journal.pone.0360407)
Supplement: Supplementary file 13 [file pone.0360407.s013.pdf]

S1 Table. Methodological comparison of image- and lexicon-based word–color frameworks.

Comparison includes prior approaches whose data, tools, or precomputed embeddings have been made publicly available, alongside the present framework. Lindner, Bonnier, & Süsstrunk (2012) [1] is the methodologically closest image-based predecessor but is not tabulated here because its underlying dataset was not released.

**Approach**	**Method type**	**Color mapping**	**Word coverage**	**Public data**	**Distinction from the present framework**
Setlur & Stone (2016) [2]	Linguistic-based (linguistic resources + color database)	1:1 single representative color, or small palette	Extensible (arbitrary words/phrases)	Algorithm fully specified; built on public resources (Google n-grams, XKCD, WordNet); no official code release	A single representative color discards the distributional structure of a word's color associations and provides no additive base-color basis, so vectors cannot be summed across words. The present framework outputs a 10-dimensional additive base-color vector that preserves proportions and natively aggregates over corpora. **Key validation (vs. the present framework):** Setlur's full-pipeline RGB output (WordNet lexical lookup, with a Bing-Image-Search k-means + W3C-nearest fallback for words not covered by the lexical step) is mapped to our 10 base-color bins and compared by top-1 agreement. Sample: 100 random English words; 17 covered by the lexical step alone (top-1 7/17, 41%), and 94 evaluable under the full pipeline (6 returned no usable images and are excluded). Full-pipeline top-1 agreement: 31/94 (33%); per-color one-vs-rest AUC median = 0.63 (S9 Fig).
Guilbeault et al. (2020) / comp-syn [3, 4]	Image-based (Google Images; perceptually uniform JzAzBz)	1:N continuous color distribution (8-d JzAzBz histogram)	∼40,000 English words (precomputed release; original study analyzes 78 WordNet-derived categories)	Yes (precomputed embeddings released)	The continuous JzAzBz distribution is not expressed in interpretable base-color units, hindering vector summation and corpus-level aggregation. The present framework adds a 10-d additive base-color representation on the same web-image + perceptually-uniform-color premise and operates at corpus scale (a poet's œuvre, the top-100 firms). The most directly comparable predecessor. **Key validation (vs. the present framework):** because comp-syn's 8-d JzAzBz octants and our 10-d CIELch base colors live in different geometries, a Representational Similarity Analysis (RSA) is used — each space's matrix of pairwise cosines over the same word sample is computed and the two matrices' upper triangles are correlated. Across 100 random samples of 100 words each: median Pearson $r = 0.458$ between the pairwise cosines (mean 0.460, SD 0.058; median Spearman $\rho = 0.421$), with all 100/100 draws above the row-shuffled null maximum (S10 Fig).

Continued on next page

S1 Table (continued from previous page)

Approach	Method type	Color mapping	Word coverage	Public data	Distinction from the present framework
NRC Word–Colour Lexicon [5]	Survey-based (Mechanical Turk; 11-color forced choice)	1:1 single top color per word	Fixed (14,000+ predefined; new words require additional surveys)	Yes (NRC PURL)	Survey-based extension to new words requires human labor; the 1:1 mapping collapses a word's multi-color profile to a single label, capturing symbolic association rather than the visual footprint. The present framework auto-aggregates pixels from the Google-indexed visual footprint, yielding 1:N additive vectors with immediate vocabulary extensibility. **Key validation (vs. the present framework):** each of our 10 base-color proportions is treated as a per-color score; per-color one-vs-rest AUC is computed over a 100-word random sample. Median per-color AUC = 0.65 across 10 base colors; top-3 chromatic accuracy = 0.680 (chance 0.429; S7 Fig).
Rathore et al. (2019) [6]	Image-based (Google Images; CIELAB tolerance-region features)	1:N association ratings over a fixed target palette (UW-58 / BCP-37)	Extensible in principle, but the model is trained on human ratings (12 fruit concepts) and estimates ratings for a predefined target color set	Yes (code and human-ratings data released)	The output is a set of association ratings over a fixed target palette rather than proportions over named base colors, and the estimation model requires supervised training on human ratings; neither supports direct corpus-level aggregation. The present framework needs no human training data and emits additive 10-d named base-color vectors that sum across corpora. **Key validation (vs. the present framework):** for each of 18 fruit/material concepts on the UW-58/BCP-37 palettes, the present framework's per-word vector is compared with Rathore's slider-rated distribution by cosine similarity. Mean per-concept cosine = 0.853 (chromatic block 0.777, achromatic block 0.920; Pearson $r = 0.656$; S8 Fig).
**Present framework** (Ours)	Image-based (Google Images; perceptually uniform CIELch)	1:N additive 10-d vector over 7 chromatic + 3 achromatic base colors	Extensible (arbitrary words; corpus-level via vector summation)	Yes (full code and processed outputs released; see SI File)	Perceptually-uniform color space, interpretable additive base-color basis, native corpus-level aggregation, and external validation against the NRC lexicon [5], Rathore et al. (2019) [6], comp-syn [3, 4], and Setlur & Stone [2]. **Key validation:** validated against the four external resources above (S7–S10 Figs); alignment method per benchmark is shown in the corresponding rows.

References

1. Lindner A, Bonnier N, Süsstrunk S. What is the Color of Chocolate? – Extracting Color Values of Semantic Expressions. In: Proceedings of the 6th European Conference on Colour in Graphics, Imaging, and Vision (CGIV 2012). Amsterdam, The Netherlands: Society for Imaging Science and Technology; 2012. p. 355-61.
2. Setlur V, Stone MC. A linguistic approach to categorical color assignment for data visualization. IEEE Transactions on Visualization and Computer Graphics. 2016;22(1):698-707. http://dx.doi.org/10.1109/TVCG.2015.2467471 doi:10.1109/TVCG.2015.2467471.
3. Guilbeault D, Nadler EO, Chu M, Lo Sardo DR, Kar AA, Desikan BS. Color associations in abstract semantic domains. Cognition. 2020;201:104306. http://dx.doi.org/10.1016/j.cognition.2020.104306 doi:10.1016/j.cognition.2020.104306.
4. Srinivasa Desikan B, Hull T, Nadler EO, Guilbeault D, Kar AA, Chu M, et al. comp-syn: Perceptually Grounded Word Embeddings with Color. In: Proceedings of the 28th International Conference on Computational Linguistics (COLING). International Committee on Computational Linguistics; 2020. p. 1744-53. http://dx.doi.org/10.18653/v1/2020.coling-main.154 doi:10.18653/v1/2020.coling-main.154.
5. Mohammad S. Colourful language: Measuring word-colour associations. arXiv preprint arXiv:13095942. 2013.
6. Rathore R, Leggon Z, Lessard L, Schloss KB. Estimating color-concept associations from image statistics. IEEE transactions on visualization and computer graphics. 2019;26(1):1226-35.
7. Google. Google Books / Google Digital Copy;. Public-domain works hosted as digital copies. https://books.google.com/.
8. Lowell A. Legends. Houghton Mifflin; 1921.
9. Internet Archive. Internet Archive — Digital Library of Free & Borrowable Texts;. Accessed for public-domain literary texts. https://archive.org/.
10. Lowell A. Can Grande's castle. Macmillan; 1918.
11. Lowell A. Pictures of the floating world. Macmillan; 1919.
12. Project Gutenberg; 2024. https://www.gutenberg.org.
13. Lowell A. A Dome of Many-Coloured Glass. ReadHowYouWant. com; 1912.
14. Lowell A. Sword Blades and Poppy Seed. ReadHowYouWant. com; 1921.
15. Lowell A. Men, women and ghosts. IndyPublish. com; 1916.
16. Johnson GD. The selected works of Georgia Douglas Johnson. G.K. Hall; 1997.
