# Supplementary material for: Word-color association from large-scale online data: Methodology and applications
Source: PLoS One. 2026 Oct 16;21(10):e0360407. doi: 10.1371/journal.pone.0360407 (PMC13674752; doi:10.1371/journal.pone.0360407)
Supplement: Supplementary file 14 [file pone.0360407.s014.pdf]

**S2 Table. Data Sources for Lowell's and Johnson's Poetry.**
Listed are the publication titles and corresponding data sources for each author.

| Movement | Author | Data Source | Title |
|---|---|---|---|
| Imagism | Amy Lowell | Google Digital Copy [7] | *Legends [8]* |
| | | Internet Archive [9] | *Can Grande's Castle [10]* |
| | | | *Pictures of the Floating World [11]* |
| | | Project Gutenberg [12] | *A Dome of Many-Coloured Glass [13]* |
| | | | *Sword Blades and Poppy Seed [14]* |
| | | | *Men, Women and Ghosts [15]* |
| Harlem Renaissance | Georgia Douglas Johnson | Internet Archive [9] | *The Selected Works of Georgia Douglas Johnson [16]* |

# References


1. Lindner A, Bonnier N, Süsstrunk S. What is the Color of Chocolate? – Extracting Color Values of Semantic Expressions. In: Proceedings of the 6th European Conference on Colour in Graphics, Imaging, and Vision (CGIV 2012). Amsterdam, The Netherlands: Society for Imaging Science and Technology; 2012. p. 355-61.
2. Setlur V, Stone MC. A linguistic approach to categorical color assignment for data visualization. IEEE Transactions on Visualization and Computer Graphics. 2016;22(1):698-707. http://dx.doi.org/10.1109/TVCG.2015.2467471 doi:10.1109/TVCG.2015.2467471.
3. Guilbeault D, Nadler EO, Chu M, Lo Sardo DR, Kar AA, Desikan BS. Color associations in abstract semantic domains. Cognition. 2020;201:104306. http://dx.doi.org/10.1016/j.cognition.2020.104306 doi:10.1016/j.cognition.2020.104306.
4. Srinivasa Desikan B, Hull T, Nadler EO, Guilbeault D, Kar AA, Chu M, et al. comp-syn: Perceptually Grounded Word Embeddings with Color. In: Proceedings of the 28th International Conference on Computational Linguistics (COLING). International Committee on Computational Linguistics; 2020. p. 1744-53. http://dx.doi.org/10.18653/v1/2020.coling-main.154 doi:10.18653/v1/2020.coling-main.154.
5. Mohammad S. Colourful language: Measuring word-colour associations. arXiv preprint arXiv:13095942. 2013.
6. Rathore R, Leggon Z, Lessard L, Schloss KB. Estimating color-concept associations from image statistics. IEEE transactions on visualization and computer graphics. 2019;26(1):1226-35.
7. Google. Google Books / Google Digital Copy;. Public-domain works hosted as digital copies. https://books.google.com/.
8. Lowell A. Legends. Houghton Mifflin; 1921.
9. Internet Archive. Internet Archive — Digital Library of Free & Borrowable Texts;. Accessed for public-domain literary texts. https://archive.org/.
10. Lowell A. Can Grande's castle. Macmillan; 1918.
11. Lowell A. Pictures of the floating world. Macmillan; 1919.
12. Project Gutenberg; 2024. https://www.gutenberg.org.
13. Lowell A. A Dome of Many-Coloured Glass. ReadHowYouWant. com; 1912.
14. Lowell A. Sword Blades and Poppy Seed. ReadHowYouWant. com; 1921.
15. Lowell A. Men, women and ghosts. IndyPublish. com; 1916.
16. Johnson GD. The selected works of Georgia Douglas Johnson. G.K. Hall; 1997.
