# Supplementary material for: Word-color association from large-scale online data: Methodology and applications
Source: PLoS One. 2026 Oct 16;21(10):e0360407. doi: 10.1371/journal.pone.0360407 (PMC13674752; doi:10.1371/journal.pone.0360407)
Supplement: Supplementary file 15 [file pone.0360407.s015.pdf]

S3 Table. Brands grouped by industry sector.

Industry Sector	Brands
Commercial services	Visa Inc., Mastercard Incorporated, S&P Global Inc.
Communications	T-Mobile US, Inc., China Mobile Ltd, AT&T Inc., Verizon Communications Inc., Deutsche Telekom AG
Consumer durables	Tesla, Inc.
Consumer non-durables	Procter & Gamble Company, LVMH, Hermès International, Kweichow Moutai Co., Ltd., Philip Morris International Inc., Nestlé S.A., PepsiCo, Inc., L'Oréal
Consumer services	McDonald's Corporation, Walt Disney Company, Booking Holdings Inc.
Electronic technology	Apple Inc., NVIDIA Corporation, Broadcom Inc., Taiwan Semiconductor Manufacturing (TSMC), ASML Holding, Cisco Systems, Inc., Samsung Electronics, GE Aerospace, Qualcomm Incorporated, Advanced Micro Devices, Inc. (AMD), Arm Holdings plc, RTX Corporation, Texas Instruments Incorporated, Boston Scientific Corporation
Energy minerals	Saudi Arabian Oil Co. (Aramco), Exxon Mobil Corporation, Chevron Corporation, PetroChina Company Limited, Shell plc, Reliance Industries Limited
Finance	Berkshire Hathaway Inc., JPMorgan Chase & Co., Bank of America Corporation, Industrial and Commercial Bank of China Limited, Wells Fargo & Company, Agricultural Bank of China Limited, Morgan Stanley, American Express Company, China Construction Bank Corporation, Blackstone Inc., Goldman Sachs Group, Inc., Bank of China Limited, HSBC Holdings plc, Commonwealth Bank of Australia, Royal Bank of Canada, BlackRock, Inc., China Merchants Bank Co., Ltd.
Health & Medical	Eli Lilly and Company, UnitedHealth Group Incorporated, Novo Nordisk A/S, Johnson & Johnson, AbbVie Inc., Roche Holding AG, International Holding Company PJSC, Abbott Laboratories, AstraZeneca plc, Merck & Company, Inc., Intuitive Surgical, Inc., Thermo Fisher Scientific Inc., Novartis AG, Amgen Inc.
Process industries	Linde plc
Producer manufacturing	Toyota Motor Corporation, Caterpillar Inc., Siemens AG, Contemporary Amperex Technology Co. Ltd. (CATL)
Retail trade	Amazon.com, Inc., Walmart Inc., Costco Wholesale Corporation, Home Depot, Inc., Alibaba Group Holding Limited, Inditex (Industria de Diseño Textil, S.A.), PDD Holdings Inc.
Technology services	Microsoft Corporation, Alphabet Inc., Meta Platforms, Inc., Oracle Corporation, Tencent Holdings Limited, Netflix, Inc., SAP SE, Salesforce, Inc., Palantir Technologies Inc., Accenture plc, IBM (International Business Machines Corporation), ServiceNow, Inc., Adobe Inc., Tata Consultancy Services Limited, Intuit Inc., Shopify Inc.
Transportation	Uber Technologies, Inc.
