# Supplementary material for: Word-color association from large-scale online data: Methodology and applications
Source: PLoS One. 2026 Oct 16;21(10):e0360407. doi: 10.1371/journal.pone.0360407 (PMC13674752; doi:10.1371/journal.pone.0360407)
Supplement: Supplementary file 16 [file pone.0360407.s016.pdf]

S4 Table. Cosine similarity between official logos and associated web images for the top 100 global companies.

Company	Cosine similarity
Roche Holding AG	0.95663
Salesforce, Inc.	0.94968
AT&T Inc.	0.94648
American Express Company	0.94307
SAP SE	0.92817
International Holding Company PJSC	0.92038
McDonald's Corporation	0.90473
IBM (International Business Machines Corporation)	0.90405
Home Depot, Inc.	0.88940
NVIDIA Corporation	0.88780
Intuit Inc.	0.88024
Costco Wholesale Corporation	0.87997
Royal Bank of Canada	0.87974
Cisco Systems, Inc.	0.87856
Walmart Inc.	0.87743
T-Mobile US, Inc.	0.87033
Shell plc	0.86898
Meta Platforms, Inc.	0.86478
Chevron Corporation	0.86291
Arm Holdings plc	0.86081
Wells Fargo & Company	0.86047
Alibaba Group Holding Limited	0.85978
Abbott Laboratories	0.84615
Mastercard Incorporated	0.82934
Caterpillar Inc.	0.82826
Novartis AG	0.82679
Procter & Gamble Company	0.82332
Samsung Electronics	0.82297
Adobe Inc.	0.82124
China Construction Bank Corporation	0.81986
PDD Holdings Inc.	0.81804
Netflix, Inc.	0.81803
Hermès International	0.81478
Linde plc	0.80083
Tesla, Inc.	0.79807
Exxon Mobil Corporation	0.79590
China Mobile Ltd	0.79363
Bank of America Corporation	0.78523
Tencent Holdings Limited	0.77266
Deutsche Telekom AG	0.77101
Commonwealth Bank of Australia	0.76979
Booking Holdings Inc.	0.75113
Qualcomm Incorporated	0.74782
PetroChina Company Limited	0.74442
Saudi Arabian Oil Co. (Aramco)	0.73756
Shopify Inc.	0.73603
S&P Global Inc.	0.73175
Verizon Communications Inc.	0.73097
Eli Lilly and Company	0.72416
PepsiCo, Inc.	0.71709

Company	Cosine similarity
Boston Scientific Corporation	0.71348
UnitedHealth Group Incorporated	0.70531
Amgen Inc.	0.70473
Agricultural Bank of China Limited	0.70414
Johnson & Johnson	0.69911
Contemporary Amperex Technology Co. Ltd. (CATL)	0.68929
Taiwan Semiconductor Manufacturing (TSMC)	0.68770
Advanced Micro Devices, Inc. (AMD)	0.67745
Palantir Technologies Inc.	0.66971
Texas Instruments Incorporated	0.66239
Philip Morris International Inc.	0.65776
Siemens AG	0.62859
Kweichow Moutai Co., Ltd.	0.61828
Microsoft Corporation	0.60434
HSBC Holdings plc	0.59101
China Merchants Bank Co., Ltd.	0.58836
Merck & Company, Inc.	0.55499
Alphabet Inc.	0.54784
Thermo Fisher Scientific Inc.	0.54488
Broadcom Inc.	0.53889
Uber Technologies, Inc.	0.52549
Toyota Motor Corporation	0.52519
Intuitive Surgical, Inc.	0.50990
BlackRock, Inc.	0.50551
Industrial and Commercial Bank of China Limited	0.50377
Accenture plc	0.49208
Blackstone Inc.	0.48346
AstraZeneca plc	0.46543
JPMorgan Chase & Co.	0.44309
Oracle Corporation	0.41190
Reliance Industries Limited	0.40745
Bank of China Limited	0.40667
Apple Inc.	0.36787
Morgan Stanley	0.36536
Amazon.com, Inc.	0.35282
ServiceNow, Inc.	0.32888
GE Aerospace	0.32619
Visa Inc.	0.29358
Berkshire Hathaway Inc.	0.27650
L'Oréal	0.27556
Novo Nordisk A/S	0.26674
Goldman Sachs Group, Inc.	0.25982
RTX Corporation	0.25672
ASML Holding	0.25489
LVMH	0.22295
AbbVie Inc.	0.22293
Tata Consultancy Services Limited	0.20799
Inditex (Industria de Diseño Textil, S.A.)	0.19526
Walt Disney Company	0.14860
Nestlé S.A.	0.11811
