# Supplementary material for: Word-color association from large-scale online data: Methodology and applications
Source: PLoS One. 2026 Oct 16;21(10):e0360407. doi: 10.1371/journal.pone.0360407 (PMC13674752; doi:10.1371/journal.pone.0360407)
Supplement: Supplementary file 17 [file pone.0360407.s017.zip › SI File/website/templates/index.html]

Word Color Association


# Word Color Association

Enter a word:


Check Text:

 True

 False

Submit
{# 상단 4개 플롯만 기준으로 표시 여부 결정 #}
{% if plot\_html\_1 %}

Scatter Plot and Histogram of Hue

{{ plot\_html\_1|safe }}

{{ plot\_html\_2|safe }}

Scatter Plot and Histogram of Lightness

{{ plot\_html\_3|safe }}

{{ plot\_html\_4|safe }}

Color Ratios

{% if plot\_html\_5 %}

{{ plot\_html\_5|safe }}

{% endif %}
{% if plot\_html\_6 %}

{{ plot\_html\_6|safe }}

{% endif %}

{% endif %}
