# Supplementary material for: Word-color association from large-scale online data: Methodology and applications
Source: PLoS One. 2026 Oct 16;21(10):e0360407. doi: 10.1371/journal.pone.0360407 (PMC13674752; doi:10.1371/journal.pone.0360407)
Supplement: Supplementary file 17 [file pone.0360407.s017.zip › SI File/csv/revision_results/polysemy.pdf]

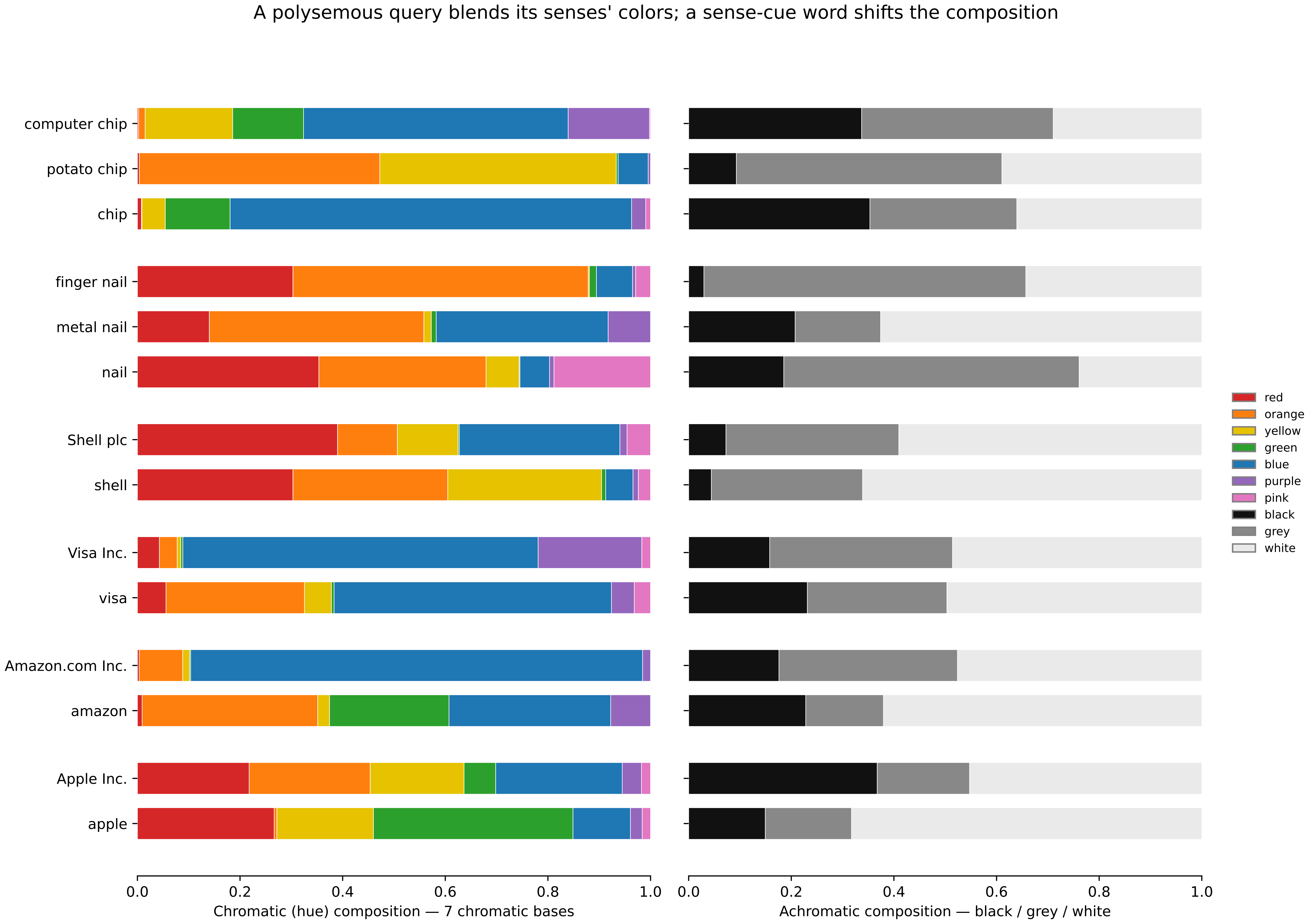
A polysemous query blends its senses' colors; a sense-cue word shifts the composition
computer chip
potato chip
chip
finger nail
metal nail
nail
Shell plc
shell
Visa Inc.
visa
Amazon.com Inc.
amazon
Apple Inc.
apple
0.0
0.2
0.4
0.6
0.8
1.0
Chromatic (hue) composition — 7 chromatic bases
Achromatic composition — black / grey / white
red
orange
yellow
green
blue
purple
pink
black
grey
white
