# Supplementary material for: Word-color association from large-scale online data: Methodology and applications
Source: PLoS One. 2026 Oct 16;21(10):e0360407. doi: 10.1371/journal.pone.0360407 (PMC13674752; doi:10.1371/journal.pone.0360407)
Supplement: Supplementary file 17 [file pone.0360407.s017.zip › SI File/csv/revision_results/page_compare.pdf]

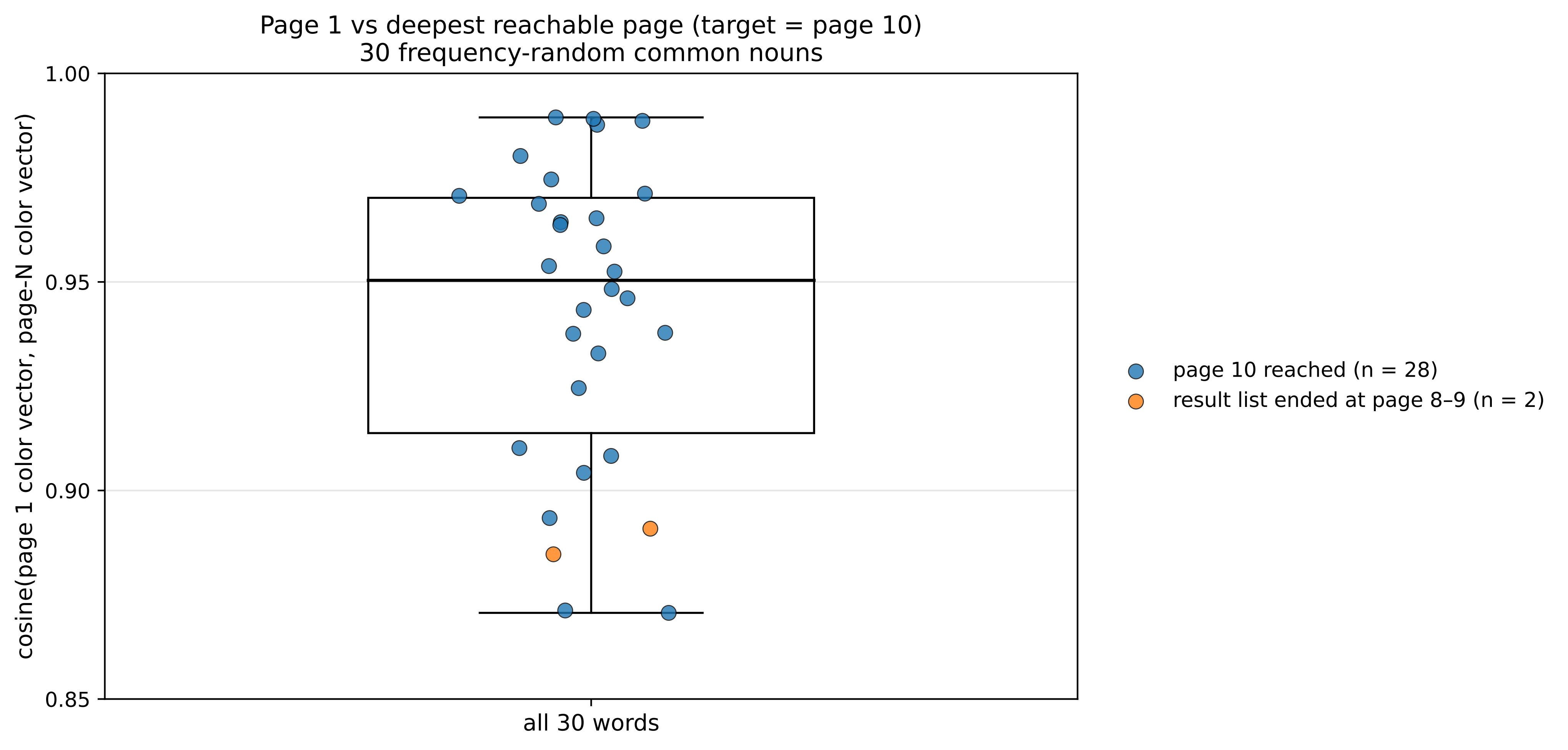
Page 1 vs deepest reachable page (target = page 10)
30 frequency-random common nouns
cosine(page 1 color vector, page-N color vector)
1.00
0.95
0.90
0.85
all 30 words
page 10 reached (n = 28)
result list ended at page 8–9 (n = 2)
