# Supplementary material for: Word-color association from large-scale online data: Methodology and applications
Source: PLoS One. 2026 Oct 16;21(10):e0360407. doi: 10.1371/journal.pone.0360407 (PMC13674752; doi:10.1371/journal.pone.0360407)
Supplement: Supplementary file 17 [file pone.0360407.s017.zip › SI File/csv/revision_results/rathore_compare.pdf]

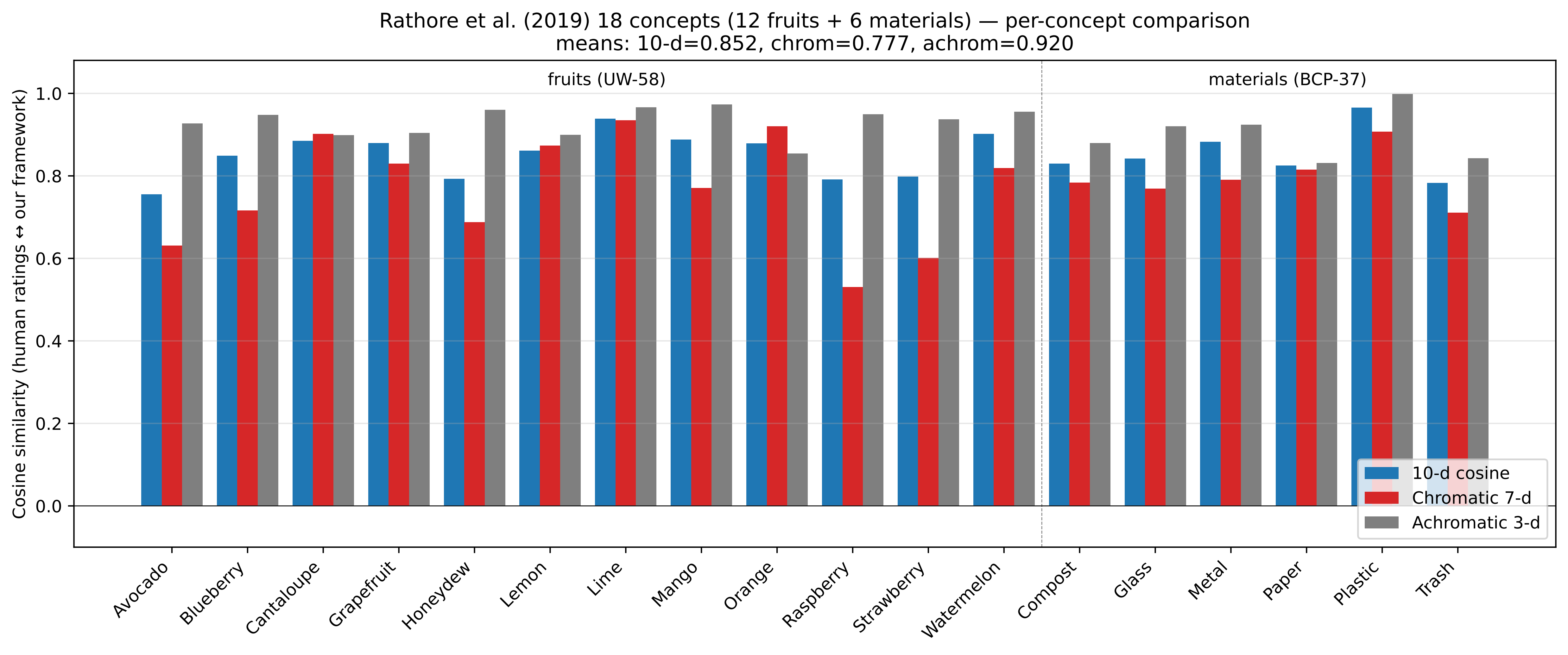
Rathore et al. (2019) 18 concepts (12 fruits + 6 materials) — per-concept comparison
means: 10-d=0.852, chrom=0.777, achrom=0.920
fruits (UW-58)
materials (BCP-37)
Cosine similarity (human ratings ↔ our framework)
1.0
0.8
0.6
0.4
0.2
0.0
10-d cosine
Chromatic 7-d
Achromatic 3-d
Avocado
Blueberry
Cantaloupe
Grapefruit
Honeydew
Lemon
Lime
Mango
Orange
Raspberry
Strawberry
Watermelon
Compost
Glass
Metal
Paper
Plastic
Trash
