# Supplementary material for: Word-color association from large-scale online data: Methodology and applications
Source: PLoS One. 2026 Oct 16;21(10):e0360407. doi: 10.1371/journal.pone.0360407 (PMC13674752; doi:10.1371/journal.pone.0360407)
Supplement: Supplementary file 17 [file pone.0360407.s017.zip › SI File/csv/revision_results/compsyn_compare_single.pdf]

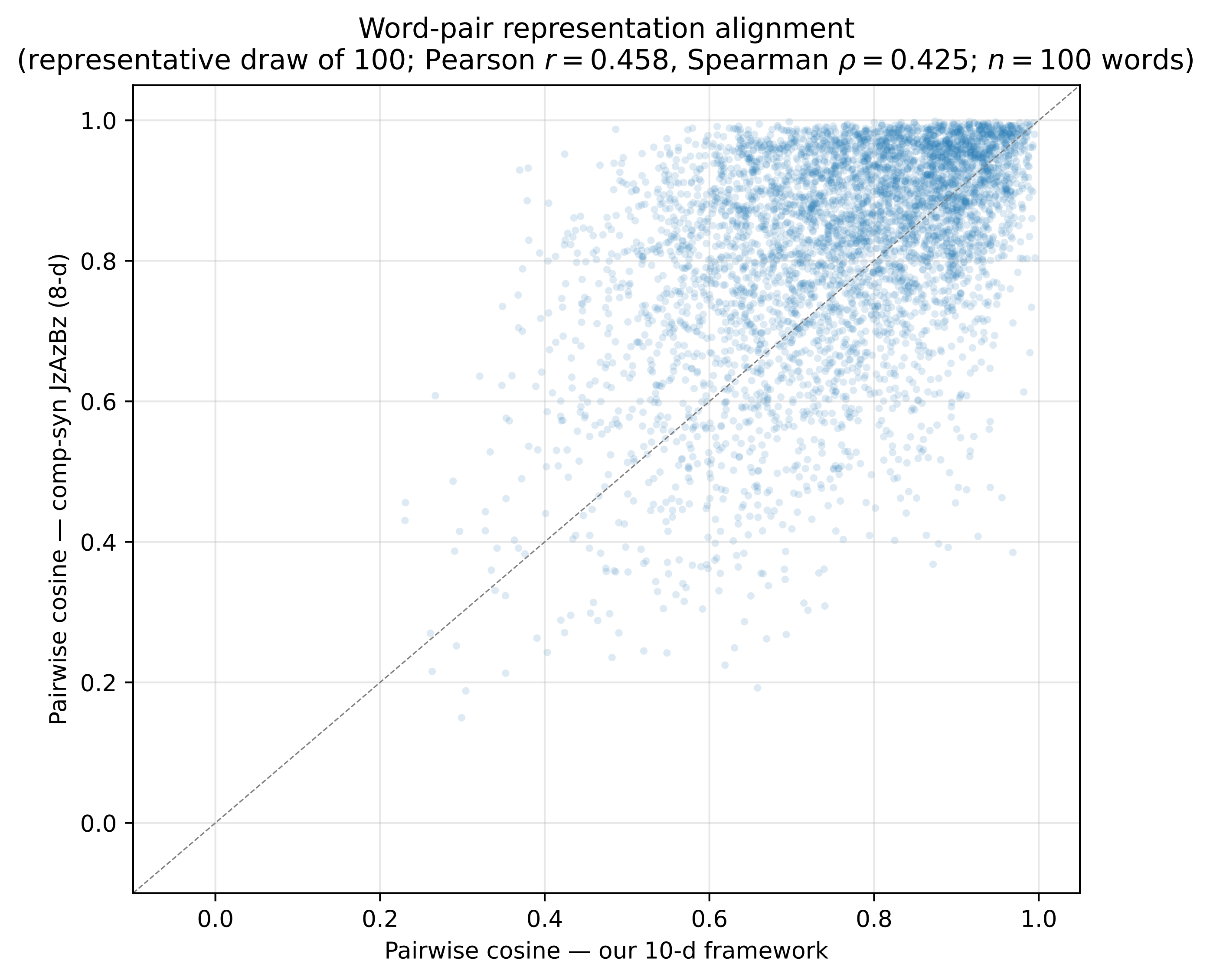
Word-pair representation alignment
(representative draw of 100; Pearson $r = 0.458$, Spearman $\rho = 0.425$; $n = 100$ words)
Pairwise cosine — comp-syn JzAzBz (8-d)
Pairwise cosine — our 10-d framework
0.0
0.2
0.4
0.6
0.8
1.0
