# Supplementary material for: Word-color association from large-scale online data: Methodology and applications
Source: PLoS One. 2026 Oct 16;21(10):e0360407. doi: 10.1371/journal.pone.0360407 (PMC13674752; doi:10.1371/journal.pone.0360407)
Supplement: Supplementary file 17 [file pone.0360407.s017.zip › SI File/data_external/nrc_color_v0.92.txt]

404 Error


# Sorry, this page doesn't exist. Please check the URL or go back a page.

## 404 Error. Page Not Found.
